# Understanding, Mimicking, and Mitigating Radiolytic Damage to Polymers in Liquid Phase Transmission Electron Microscopy

**DOI:** 10.1002/adma.202402987

**Published:** 2024-11-16

**Authors:** Hanglong Wu, Hongyu Sun, Roy A. J. F. Oerlemans, Siyu Li, Jingxin Shao, Jianhong Wang, Rick R. M. Joosten, Xianwen Lou, Yingtong Luo, Hongkui Zheng, Loai K. E. A. Abdelmohsen, H. Hugo Pérez Garza, Jan C. M. van Hest, Heiner Friedrich

**Affiliations:** ^1^ Bio‐Organic Chemistry Institute for Complex Molecular Systems Eindhoven University of Technology P.O. Box 513 Eindhoven 5600 MB The Netherlands; ^2^ Center for Multiscale Electron Microscopy Department of Chemical Engineering and Chemistry Eindhoven University of Technology Eindhoven 5600 MB The Netherlands; ^3^ DENSsolutions B.V. Informaticalaan 12 Delft 2628 ZD The Netherlands; ^4^ Laboratory of Physical Chemistry Department of Chemical Engineering and Chemistry Eindhoven University of Technology Eindhoven 5600 MB The Netherlands

**Keywords:** in situ TEM, liquid‐phase electron microscopy, polymers, radiation damage, radical scavengers

## Abstract

Advances in liquid phase transmission electron microscopy (LP‐TEM) have enabled the monitoring of polymer dynamics in solution at the nanoscale, but radiolytic damage during LP‐TEM imaging limits its routine use in polymer science. This study focuses on understanding, mimicking, and mitigating radiolytic damage observed in functional polymers in LP‐TEM. It is quantitatively demonstrated how polymer damage occurs across all conceivable (LP‐)TEM environments, and the key characteristics and differences between polymer degradation in water vapor and liquid water are elucidated. Importantly, it is shown that the hydroxyl radical‐rich environment in LP‐TEM can be approximated by UV light irradiation in the presence of hydrogen peroxide, allowing the use of bulk techniques to probe damage at the polymer chain level. Finally, the protective effects of commonly used hydroxyl radical scavengers are compared, revealing that the effectiveness of graphene's protection is distance‐dependent. The work provides detailed methodological guidance and establishes a baseline for polymer degradation in LP‐TEM, paving the way for future research on nanoscale tracking of shape transitions and drug encapsulation of polymer assemblies in solution.

## Graphical abstract

This work addresses radiolytic damage to polymers in liquid phase transmission electron microscopy (LP‐TEM), which restricts its routine use in soft matter science. It quantifies polymer degradation behaviors across all conceivable (LP‐)TEM environments, mimics beam‐induced damage using UV light and hydrogen peroxide, evaluates hydroxyl radical scavengers and proposes effective strategies for damage mitigation.

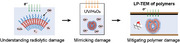

## 1 Introduction

Understanding how polymers in liquid media self‐assemble into vesicles, interact with each other and respond to different environmental stimuli (e.g., pH and temperature) is important for the design of drug delivery vehicles, micro/nanoreactors, and artificial systems with life‐like behaviors.^[^ ^1^ ^]^ Time‐resolved cryogenic transmission electron microscopy (Cryo‐TEM) has proven to be a powerful tool for studying the morphological stages of polymer assembly over time and providing 3D structural information.^[^ ^2^ ^]^ However, it fails to offer insight into the evolution of individual polymer nanoparticles during self‐assembly or interaction processes.^[^ ^2^, ^3^ ^]^ Recent advances in liquid‐phase transmission electron microscopy (LP‐TEM) allow us to track the evolution of polymeric materials in a variety of solvents with a unique combination of sub‐second temporal resolution and nanometer spatial resolution.^[^ ^2^, ^4^ ^]^ To date, LP‐TEM has successfully been used to study a broad range of both natural and synthetic polymer systems, including DNA,^[^ ^5^ ^]^ peptides,^[^ ^6^ ^]^ proteins,^[^ ^7^ ^]^ single polymer chains,^[^ ^8^ ^]^ and supramolecular assemblies such as coacervates, micelles, and vesicles.^[^ ^9^ ^]^ Despite these tremendous achievements, radiolytic damage caused by the formation of reactive radicals from electron beam‐induced radiolysis of water remains a major obstacle to the routine use of LP‐TEM in polymer studies.^[^ ^10^ ^]^ The achievable spatial resolution is also inherently limited by the beam sensitivity of the polymer sample. During LP‐TEM imaging with ionizing radiation, polymers are inevitably prone to undergo rapid cross‐linking and degradation,^[^ ^4^, ^10^ ^]^ and as a result, it is usually difficult, and sometimes even impossible to completely decouple native polymer dynamics from artifacts induced by radiolytic damage in LP‐TEM data.^[^ ^4^, ^10^, ^11^ ^]^ The key to solving this problem is, therefore, not only to mitigate but to fundamentally understand radiolytic damage to polymers in LP‐TEM.^[^ ^4^, ^10^, ^12^ ^]^

To this end, it is crucial to investigate the critical dose limits tolerable by the polymer and the liquid medium under study, including both the electron flux thresholds and total dose thresholds,^[^ ^3^, ^4^, ^9^, ^10^ ^]^ and to understand the radiolytic environment inside the liquid cell.^[^ ^4^, ^10^, ^12^ ^]^ The critical irradiation thresholds are typically established by comparing the structure and dynamics of samples at various flux and dose conditions and have been more accurately determined by correlating LP‐TEM with other complementary techniques, such as fluorescence microscopy,^[^ ^13^ ^]^ and post‐mortem mass spectrometry.^[^ ^6^, ^12^ ^]^ Unfortunately, real‐time probing of beam damage artifacts to the chemistry of polymers is not yet possible with the current instrumentation designs during LP‐TEM imaging.^[^ ^4b^ ^]^ More importantly, due to the limited sample volume (on the order of a few nanolitres) in the liquid cell, most bulk characterization tools commonly used for analyzing polymers at the vial level in the lab, such as dynamic light scattering (DLS) and gel permeation chromatography (GPC), cannot be directly applied to examine the samples in LP‐TEM. Fortunately, the aqueous radiolytic environment in the liquid cell, including the formed primary radicals and their steady‐state concentrations, can now be estimated by using radiation chemistry simulations.^[^ ^10^, ^12^, ^14^ ^]^ Thus, it is theoretically possible to replicate a similar solution environment in a vial to mimic the radiolytic damage to polymers observed in LP‐TEM on a macroscopic scale. This approach would allow, for example, the measurement of molecular weight changes in polymer chains caused by radiolytic chain cleavage or cross‐linking. However, the LP‐TEM community currently lacks such comprehensive methods to bridge the gap between radiation chemistry in the liquid cell and the reactions occurring in bulk solution.^[^ ^12^, ^15^ ^]^

In the realm of LP‐TEM, the use of scavengers, either in liquid or solid form, has become increasingly popular to mitigate radiolytic damage when imaging polymers.^[^ ^12^, ^16^ ^]^ Among the most commonly used scavengers, isopropanol alcohol (IPA) and graphene have been well documented for their effectiveness in scavenging hydroxyl radicals (OH^•^), which are known to be highly detrimental to most polymers during LP‐TEM experiments.^[^ ^4^, ^12^, ^16^ ^]^ However, whether the presence of scavengers might alter the intrinsic behaviors of polymers, as typically observed in vitro or in vivo, remains an unexplored and unclear aspect of this research.^[^ ^4a^ ^]^ Additionally, despite the extensive use of scavengers in LP‐TEM thus far, there is a lack of fundamental studies on their applicability in imaging functional polymer assemblies in the liquid cell. Such studies are critical, as they could provide key insights into, for example, drug encapsulation and release processes, with significant implications for polymer science and biomedical research. Another essential aspect yet to be explored is the role of spatial proximity between the sample and graphene in LP‐TEM. Understanding how this distance influences graphene's ability to protect against hydroxyl radicals is paramount for the future design of graphene‐based liquid cells.^[^ ^4^, ^16^ ^]^

In this work, we focus on understanding, mimicking, and mitigating the radiolytic damage to functional polymers in LP‐TEM. Due to their high relevance for biomedical applications, we selected bowl‐shaped poly(ethylene glycol)‐*b*‐polystyrene (PEG‐*b*‐PS) vesicles, or stomatocytes, in aqueous solution as our primary model system. Furthermore, these stomatocytes have been widely studied by Cryo‐TEM and PEG‐*b*‐PS exhibits relatively high contrast and a robust hierarchical structure, which are ideal for LP‐TEM experiments.^[^ ^17^ ^]^ Here we describe the variation in damage pathways of these polymer vesicles trapped in different environments upon exposure to ionizing electron irradiation in LP‐TEM, including dry, water vapor, and liquid water with/without radical scavengers. We demonstrate that the oxidative radiolytic environment at low electron flux conditions (<1 e^−^ Å^−2^ s^−1^) can be recreated on a vial/bulk scale to produce similar radiolytic damage. This is achieved by using UV light irradiation in the presence of hydrogen peroxide (UV/H_2_O_2_), allowing the damage occurring at the molecular level in the block copolymer to be investigated using bulk techniques including GPC and matrix‐assisted laser desorption/ionization mass spectrometry (MALDI‐MS). Finally, we show that the hydroxyl radical scavenger graphene can protect block polymers better than IPA, but the distance between the graphene and the sample determines the degree of protection. We anticipate that this work will lead to a better and more rational design of LP‐TEM experiments on polymeric specimens.

## 2 Results and Discussion

**Figure 1:**
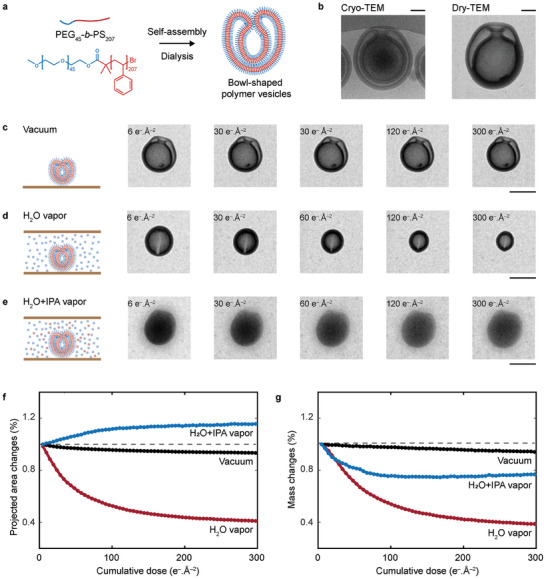
Investigating radiation damage to polymer vesicles in vacuum, H_2_O vapor, and H_2_O + IPA vapor. a) Schematic showing the synthesis of the bowl‐shaped PEG‐b‐PS polymer vesicles, i.e., stomatocytes. b) Cryo‐TEM and dry‐TEM image of a stomatocyte. Scale bars: 100 nm. c–e) Electron beam damage to polymer stomatocytes under three different environments (Movie S1, Supporting Information): c) in a vacuum, d) in H_2_O vapor, and e) in H_2_O + IPA vapor (5 v%). Scale bars: 200 nm. f,g) Image analysis of dose series showing projected area change f) and mass change g) of the stomatocyte as a function of the cumulative dose. Electron flux: 0.6 e^−^ Å^−2^ s^−1^. Scale bars: 400 nm.

Amphiphilic block copolymer PEG‐*b*‐PS stomatocytes were prepared through a well‐established protocol (Figures S1–S5, Section S3.1, Supporting Information).^[^ ^17^ ^]^ Briefly, the pre‐synthesized PEG‐*b*‐PS block copolymers were first assembled into spherical vesicles by gradually adding water to the polymer dissolved in organic solvents, which were then dialyzed against 20 mm sodium chloride (NaCl) solution to induce shape transformation into stomatocytes (**Figure** **1**a). Cryo‐TEM data confirmed the formation of bowl‐shaped polymer vesicles, or stomatocytes, with a membrane thickness of ≈25 nm and a well‐defined neck/opening size of ≈70 nm (Figure 1b; Figure S6, Supporting Information). The resulting stomatocytes are mechanically robust, maintaining their structure without significant deformation during the drying process (Figure 1b) due to the support provided by the inner membrane. Unlike spherical PEG‐b‐PS vesicles which are prone to considerable structural deformation upon drying (Figure S7, Supporting Information), the structural robustness of the stomatocytes allows for their loading into the liquid cell in either wet or dry states. Radiation damage assessment was carried out in bright‐field TEM mode using two different LP‐TEM flow holders, each featuring distinct viewing window dimensions (Sections S3.2–S3.4, Supporting Information). In some instances, custom‐made graphene‐coated silicon nitride (SiN) liquid cells were also employed. Details on the fabrication of graphene‐coated SiN cells and GLCs are given in Section S3.8 (Supporting Information). For LP‐TEM, the confinement effects within the cell must be considered. A detailed discussion is provided in Section S3.3.3 (Supporting Information). Since most polymer assemblies are amorphous, it is not possible to evaluate their structural changes induced by ionizing electron radiation in reciprocal space by electron diffraction. Therefore, the damage to the polymer structures was primarily tracked by quantifying the projected area (indicating structural deformation) and the intensity evolution (indicating mass change) of the stomatocytes as a function of the electron flux and the cumulative dose. Note that we use the unit of e^−^ Å^−2^ s^−1^ to describe the applied electron flux conditions and the corresponding dose rate in Gy/s for 200 kV electrons is provided in the main text and Table S1 (Supporting Information). For simplicity, the dose rate values presented in the main text do not account for the thickness of the liquid layer thickness, unless specified otherwise. However, the methods^[^ ^10^, ^18^ ^]^ for converting electron flux to dose rate, both with and without considering the liquid thickness effects, are detailed in Section S3.2.1 (Supporting Information). Furthermore, it is worth noting that in the literature, terms like electron exposure or electron fluence are also used in place of cumulative dose to better describe the physical principles underlying beam damage.^[^ ^6^, ^12^ ^]^

### 2.1 Investigating Radiolytic Damage to Polymers in H_2_O Vapor

To enable high‐resolution LP‐TEM imaging of samples by reducing the liquid thickness, it has recently become more routine to introduce a bubble into the SiN liquid cell through water radiolysis,^[^ ^10b^ ^]^ dissolved gas in the liquid,^[^ ^19^ ^]^ or electrochemical hydrogen evolution reaction.^[^ ^20^ ^]^ Generating a water vapor environment within the liquid cell has also enabled the investigation of fundamental processes, including water condensation and metal oxidation.^[^ ^21^ ^]^ However, water vapor radiolysis can also lead to the formation of several reactive radicals, such as hydroxyl radicals and hydrogen radicals.^[^ ^22^ ^]^ Therefore, severe radiolytic damage may also occur on hydrated samples in the vapor phase, especially considering the elevated oxygen concentration present.^[^ ^23^ ^]^ This effect should be particularly evident when imaging samples through a bubble or at the liquid‐air interface. Nevertheless, water vapor‐induced damage in LP‐TEM has usually been overlooked, and there is very limited literature on water vapor radiolysis.^[^ ^22^, ^24^ ^]^ In addition, previous radiation chemistry studies have shown that polymers in the dry state can also undergo cross‐linking and scission when subjected to electron irradiation, but such damage has rarely been considered when interpreting LP‐TEM results.^[^ ^25^ ^]^ Consequently, to build up our understanding of the radiolytic damage to polymers in LP‐TEM, we first imaged the stomatocytes in vacuum (dry) and water vapor (Figures 1c,d and Movie S1, Supporting Information) before examining them in liquid water. For completeness, we also explored the scavenging effect of IPA on polymers in the water vapor atmosphere (Figure 1e and Movie S1, Supporting Information).

Polymer stomatocytes from the same batch were exposed to electron irradiation with different electron fluxes over time 1) in the vacuum on a SiN membrane, 2) in water vapor, and 3) in water vapor + IPA inside closed liquid cells (Figure 1; Figures S8–S15, Movie S1, Supporting Information). Figure 1 mainly shows the results obtained at an electron flux of 0.6 e^−^ Å^−2^ s^−1^ for which three different processes could be observed depending on the environment. In a vacuum, there were no obvious beam‐induced morphological changes in dry stomatocytes (Figure 1c). In contrast, in water vapor at 100% relative humidity, a reduction in the projected area took place as the structure contracted (Figure 1d). We have also summarized all three possible scenarios that polymer stomatocytes could be exposed to in the water vapor environment in the liquid cell in Figure S9 (Supporting Information). We found that polymers gradually dissolved from the outside inward over time, i.e., the outer membrane dissolved first (Figure S10, Supporting Information), and as a result sometimes the stomatocyte structure could be flattened upon irradiation. Such flattening behavior can be seen more clearly in some of our vapor‐phase TEM experiments when polymers were surrounded by a thin layer of liquid water (Figure S11, Supporting Information, also see the discussion of the thin water layer in Section S3.2.2, Supporting Information). The dissolution process of these stomatocytes at the liquid‐air interface was also found to be significantly accelerated even at lower electron fluxes, presumably due to a higher concentration of hydroxyl radicals being formed in the surrounding liquid water. When water + IPA vapor was introduced into the liquid cell intended to scavenge the formed hydroxyl radicals, the polymer stomatocyte remained largely intact, and the outer membrane dissolution was prevented even when the polymer was trapped at the liquid‐air interface (Figure 1e). However, the interior bilayer membrane of the cavity still slowly disintegrated likely due to the limited accessibility of IPA to the stomatocyte's interior, which eventually led to the overall structural collapse, i.e., flattening of the stomatocyte.

We then quantified electron damage under three environmental conditions by tracking the evolution of the projected area and mass changes of stomatocytes in response to cumulative electron exposure (Figures 1f,g). Our results show that at a cumulative dose of 300 e^−^ Å^−2^, the stomatocyte area decreased by ≈7% in vacuum and by ≈60% in water vapor, while it expanded by ≈16% when exposed to IPA + water vapor (Figure 1f). This size expansion is probably due to the stomatocyte's flattening, a consequence of the dissolution of its internal membrane structures. In vacuum experiments, polymer damage was also assessed under extreme imaging conditions, revealing only a ≈12% reduction in the area after continuous imaging at 10 e^−^ Å^−2^ s^−1^ up to a cumulative dose of 5000 e^−^ Å^−2^ (Figure S8, Supporting Information). Mass changes in the stomatocyte are related to the projected intensity changes of the stomatocyte, and a contrast decrease in bright‐field TEM (BF‐TEM) is indicative of mass loss.^[^ ^26^ ^]^ Therefore, we calculated the total intensity change in the stomatocyte area to describe the mass changes that occur in the stomatocyte during the *e*‐beam irradiation (Figure 1g; Figure S12, Supporting Information). The average intensity evolution data of the stomatocyte membrane is also provided in Figure S13 (Supporting Information). Our analysis revealed that the stomatocytes exposed to irradiation in the three environments all experienced varying degrees of mass loss with ≈6% in vacuum, ≈61% in water vapor, and ≈23% in water vapor + IPA (Figure 1g and Section S3.2, Supporting Information). It's worth noting that both vacuum and water vapor experiments exhibited an initial slight intensity decrease in BF‐TEM, indicating a small degree of polymer densification before mass loss (Figure S13, Supporting Information). Considering both the area and mass loss changes due to *e*‐beam irradiation, the difference between the vacuum and water vapor results again highlights the importance of water (vapor) radiolysis in polymer damage. Furthermore, our water + IPA experiments also strongly suggest that 1) hydroxyl radicals generated by water (vapor) radiolysis are largely responsible for polymer dissolution, and 2) the introduction of IPA cannot entirely scavenge the hydroxyl radicals formed, which can still cause bond cleavage in polymers, leading to significant mass loss of the stomatocyte. Importantly, the background intensity in the vicinity of all imaged stomatocytes showed no change, indicating the absence of detectable material deposition events on the SiN membrane during polymer dissolution (Figure S14, Supporting Information). This absence of solid deposits on the SiN membrane suggests that after hydrogen abstraction (H‐abstraction) by hydroxyl radicals, the resulting reactive PEG and PS polymer chains can react rapidly with O_2_ in the water vapor to form peroxyl and oxyl radicals, which decompose via backbone scission to low molecular weight carbonyl (LMWC) compounds.^[^ ^4^, ^27^ ^]^

Based on the above data, we can conclude that at low electron fluxes and low cumulative doses, the beam‐induced damage to stomatocytes in the dry state is close to negligible. However, in water vapor, stomatocytes undergo significant mass loss likely through chain scission induced by the reaction of the polymer with hydroxyl radicals and subsequent reactions between polymer macroradicals and O_2_. Our water + IPA vapor experiments show that IPA can protect polymer vesicles to some extent, but its protective effect is limited to accessible volumes, which leaves the interior membrane of the stomatocyte unprotected.

### 2.2 Monitoring Radiolytic Damage to Polymers in Liquid Water

**Figure 2:**
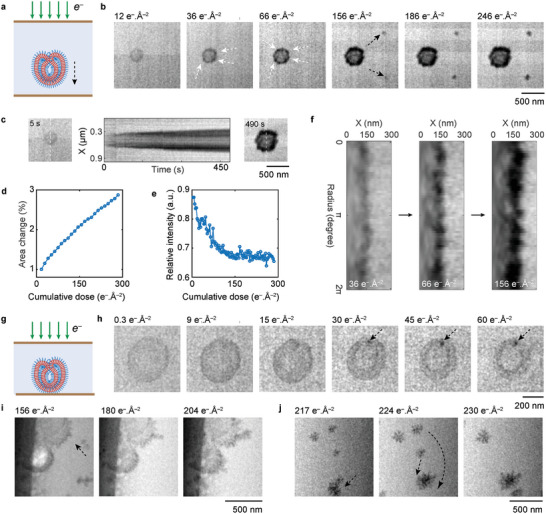
Radiolytic damage to polymer stomatocytes in liquid water. a,b) Schematic cross‐section of a liquid cell a) and 5‐frame‐average TEM image sequence (Movie S2, Supporting Information) showing a stomatocyte that is exposed to electron irradiation in a thick water layer (>1 µm). Electron flux: 0.6 e^−^ Å^−2^ s^−1^. The white arrows point to nanoparticle deposition on the stomatocyte and the black arrows indicate nanoparticle deposition outside the stomatocyte. c) Cross‐sectional time series^[^ ^9e^ ^]^ of the stomatocyte imaged at 0.6 e^−^ Å^−2^ s^−1^ with TEM image showing the first and last frame, respectively. The white vertical dashed lines indicate where the intensity line profile at each time point across the stomatocyte (middle figure) was taken. d) Projected area and e) relative intensity of the stomatocyte as a function of the cumulative dose. f) Angular maps^[^ ^9e^ ^]^ of the stomatocytes at a cumulative dose of 36, 66 and 156 e^−^ Å^−2^ showing nanoparticle deposition on the stomatocytes. g,h) Schematic cross‐section of the liquid cell (g) and TEM image sequence (h) showing a stomatocyte immobilized on the bottom window at different cumulative doses through a liquid water layer of ≈600 nm (see Movie S3, Supporting Information). Electron flux: 0.3 e^−^ Å^−2^ s^−1^. The black arrows highlight nanoparticle formation on the membrane. i) TEM image sequence showing that preformed dendritic nanoparticles attached to a damaged stomatocyte and a trapped air bubble escaped from the cavity of the stomatocyte upon electron irradiation (see Movie S4, Supporting Information). j) TEM images of an aggregation process of dendritic nanoparticles in solution where the dashed black arrows indicate the direction of nanoparticle movement between frames (see Movie S5, Supporting Information). Electron flux: 0.6 e^−^ Å^−2^ s^−1^.

To investigate the polymer damage behaviors in a liquid water environment, stomatocytes were first loaded into a liquid cell (window dimension: 30 µm × 30 µm) by employing a direct drop‐casting approach, wherein 600 nL of a stomatocyte suspension (1 mg mL^−1^) was applied onto the bottom chip before the liquid cell assembly. Note, that this drop casting approach in all 10 attempts resulted in the formation of a rather thick liquid cell (>1 µm, based on direct measurements using our earlier published thickness approximation method^[^ ^28^ ^]^) (**Figure** **2**a). Due to the large liquid layer thickness, the visibility of the stomatocytes under an electron flux of 0.6 e^−^ Å^−2^ s^−1^ was initially limited in the first few seconds. However, as the cumulative dose increased, there was a noticeable enhancement in membrane contrast and thickness (Figure 2b; Figure S16, Movie S2, Supporting Information). At a cumulative dose of 36 e^−^ Å^−2^, the formation of multiple high‐contrast patches was observed on the membrane, which eventually coalesced into a high‐contrast shell with a thickness of 90 nm. Simultaneously, nanoparticle deposition on the SiN membrane was observed. The expansion process of the stomatocyte was further elucidated through a cross‐sectional time series^[^ ^9e^ ^]^ (Figure 2c), which clearly shows nanoparticles depositing on the outer membrane, gradually forming a thick shell. By quantifying the evolution of size and pixel intensity of the stomatocyte, we identified a clear correlation between the cumulative dose and the structural evolution of the stomatocyte during LP‐TEM imaging. Specifically, the size of the stomatocyte increased in an almost linear manner with the cumulative dose (Figure 2d), indicating that the formation of the nanoparticles seems closely related to the quantity of specific water radiolysis products.^[^ ^10b^ ^]^ Regarding the intensity changes, the average intensity at the center of the stomatocyte exhibited exponential decay (Figure 2e) which could be attributed to the increased thickness of the outer shell. Furthermore, we also analyzed the local structure evolution of the membrane using the angular map time series (Figure 2f),^[^ ^9e^ ^]^ which revealed the growth of these patches and a transition in the interior structure's contrast from homogeneous to heterogeneous, probably due to the fragmentation of the membrane. Indeed, at a cumulative dose of ≈240 e^−^ Å^−2^, some stomatoctyes were completely damaged, transforming entirely into fragments (Figure S17, Supporting Information).

To gain a deeper understanding of early‐stage damage occurring on the stomatocyte, we prepared a SiN cell with a thinner liquid water layer, ≈600–700 nm in thickness. This was achieved by loading typically 1.5 nL of stomatocyte dispersion onto the liquid cell bottom chip using an automated piezoelectric‐actuated ultra‐low volume dispensing device and drying it out.^[^ ^3^, ^28^, ^29^ ^]^ Furthermore, a smaller viewing window was used (e.g., 7 µm × 12 µm) to reduce the effects of SiN membrane bulging upon filling the closed cell with liquid water (Figure S18, Supporting Information). Using this approach, the spatial resolution of LP‐TEM images was estimated to be ≈5 nm, based on the 25–75% edge width method,^[^ ^30^ ^]^ at a cumulative dose of 1.5 e^−^ Å^−2^, with an electron flux of 0.30 e^−^ Å^−2^ s^−1^ (See detailed discussion in Section S3.3.4, Supporting Information). Subsequently, the stomatocytes were subjected to electron beam exposure under similar imaging conditions (Figures 2f–i; Figures S18,  S19, Movies S3–S5, Supporting Information). In this scenario, we observed similar radiolytic damage pathways to stomatocytes as in the thick water layer experiment, including an increase in membrane contrast and thickness, the formation of patches on the membrane, and the deposition of nanoparticles on the SiN window. Notably, we found that the initial increase in membrane contrast (<9 e^−^ Å^−2^) was likely due to the formation of some nanoparticles ranging in size from 5 to 8 nm, either on top of or inside the membrane (Figure 2g). Some of these nanoparticles subsequently evolved into separate patches (30 e^−^ Å^−2^). Furthermore, we observed that the membrane growth could occur through the attachment of preformed dendritic aggregates close to the stomatocyte (Figure 2h and Movie S4, Supporting Information). These dendritic aggregates nanoparticles showed rapid movement on the SiN membrane, often aggregating into larger dendritic structures, and sometimes even forming networks (Figure 2i and Movie S5, Supporting Information). Interestingly, due to our particle loading method, air bubbles could be trapped within the cavity of some stomatocytes (Figure 2h), and the release of these bubbles indicated the rupture of the internal membrane, probably due to significant membrane fragmentation.

The observed radiolytic damage to block copolymer stomatocytes in liquid water, particularly the formation of nanoparticles and the bilayer membrane fragmentation, is significantly different from their behavior in a water vapor environment. We believe this discrepancy can be attributed to differences in oxygen and hydroxyl radical concentrations between water vapor and liquid water under the same imaging conditions. First, the gas phase typically contains a much higher concentration of oxygen compared to the equilibrium aqueous phase at room temperature. Consequently, the reactive PEG and PS polymer chains, undergoing hydrogen‐abstraction reactions, can readily react with oxygen in the water vapor, and eventually decompose into low‐molecular‐weight compounds through backbone chain scission.^[^ ^4b^ ^]^ Conversely, due to the significantly lower concentration of O_2_ in liquid water, the probability of backbone scission of these polymer chains is substantially reduced. To support our hypothesis, we exposed those nanoparticles formed in liquid water to water vapor in the liquid cell and found they were rapidly dissolved (Figure S20, Supporting Information). This observation strongly suggests the importance of the O_2_ concentration in the radiolytic damage processes of polymers. Second, liquid water is presumed to generate more hydroxyl radicals owing to its higher water molecule concentration compared to the vapor phase. As a result, the cross‐linking reactions between the polymer macroradicals in liquid water are favored, leading to the formation of insoluble polymer fragments induced by the crosslinking of PEG and PS macromolecules. Regarding the effects of flow on the radiation chemistry in our experiments, we believe the flow of aerated (O_2_‐saturated) solutions primarily contributes to the suppression of reactive atomic hydrogen and hydrated electrons through O_2_ scavenging, while continuously replenishing the O_2_ concentration.^[^ ^31^ ^]^ Under de‐aerated flow conditions, however, the effects would be reversed: the O₂ concentration would decrease, and short‐lived species such as atomic hydrogen and hydrated electrons would increase.^[^ ^31^, ^32^ ^]^

To understand how radiolytic damage occurs on organic materials in reciprocal space, we have expanded our investigation to include additional electron diffraction experiments on crystalline zeolitic imidazolate framework‐8 (ZIF‐8) nanocrystals (Figures S21–S23, Supporting Information), which can be considered as semi‐organic‐material. These experiments enabled us to monitor the structural changes of the ZIF‐8 framework under *e*‐beam irradiation in liquid, as evidenced by the intensity decay of {110} Bragg spots (Figure S23, Supporting Information). Notably, the critical doses for four measured ZIF‐8 nanoparticles were found to vary by several orders of magnitude, differing from previously reported results.^[^ ^33^ ^]^ We speculate that these discrepancies could be attributed to intrinsic crystal defects that occur during synthesis and variations in the local solution chemistry during electron irradiation. Detailed discussion is provided in Section S3.4 (Supporting Information).

To summarize, by comparing the results obtained from LP‐TEM with those obtained from vapor‐phase TEM, we have established that radiolytic damage in polymer vesicles manifests through changes in membrane thickness and contrast, and most notably, through the formation of polymer fragment nanoparticles, probably due to cross‐linking. Our finding also indicates that similar radiolytic damage occurs in the liquid water environment within the dose rate range used, with higher dose rates accelerating the damage process, likely in a sublinear manner (see detailed discussion of dose rate effects in Section S3.3.5, Supporting Information).^[^ ^10b^ ^]^ However, it should be noted that TEM data alone does not allow us to understand radiolytic damage at the level of individual polymer chains. For example, how the molecular weight changes in the PEG and PS chains remains unclear which necessitates experiments that can mimic radiolytic damage on the bulk/vial scale. To assess this process, we explored the use of standard bulk methods to analyze the progression of radiolytic damage on the molecular scale.

### 2.3 Mimicking Electron‐Induced Radiolysis of Polymers at the Vial Scale Using UV/H_2_O_2_

**Figure 3:**
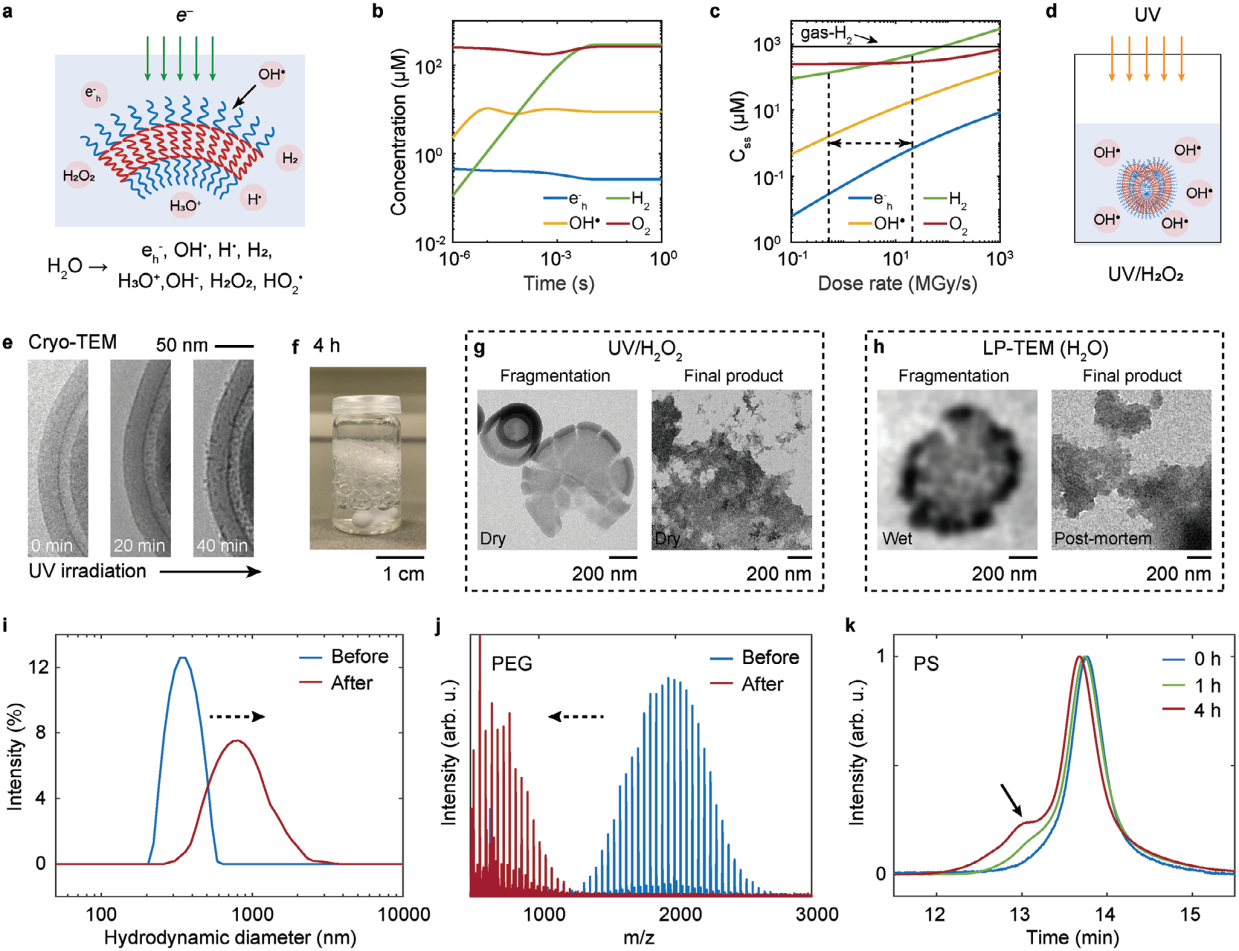
Mimicking radiolytic damage of stomatocytes in a vial using UV/H_2_O_2_. a) Schematic showing the radiolysis of water under electron beam radiation. b) Theoretical temporal evolution of four radicals generated by irradiating aerated water at 25 °C and pH 7 with an electron flux of 0.6 e^−^ Å^−2^ s^−1^ (corresponding to a dose rate of 2.68 × 10^6^ Gy s^−1^, without accounting for the effects of liquid layer thickness). c) Steady‐state concentrations (C_ss_) of species formed in aerated water as a function of dose rate. Two black dashed lines highlight the dose rate range typically used for imaging polymers in LP‐TEM. The horizontal black line indicates the theoretical solubility limit of H_2_ in H_2_O (≈8.3 × 10^2^ µm). d) Schematic showing the polymer exposed to UV in the presence of H_2_O_2_ in water. e) Cryo‐TEM images showing typical morphological changes on single PEG‐*b*‐PS stomatocytes exposed to UV/H_2_O_2_ in water over time. More Cryo‐TEM images are shown in Figure S25 (Supporting Information). f) Stable foam formation after exposing stomatocytes to UV irradiation for 4 h ([H_2_O_2_] = 10–16 wt%). g) Dry‐TEM image of the freeze‐dried sample after UV exposure. h) TEM images showing similar fragmentation and final product morphology in LP‐TEM experiments. i) DLS analysis of changes in hydrodynamic diameter of stomatocytes before and after UV exposure. j) Mass spectrum (MALDI‐MS) of PEG chains of the stomatocytes before (blue) and after (red) the UV/H_2_O_2_ experiments. k) GPC analysis of PS chains of the stomatocytes at different UV exposure times.

To investigate radiolysis‐induced chemical damage at the level of polymer chains, it is crucial to replicate the observed radiolytic damage in liquid cell experiments under controlled laboratory conditions, enabling the utilization of bulk characterization tools typically accessible only at larger flask scales. Given the widely accepted understanding that OH^•^ radicals largely contribute to polymer damage under ionization irradiation,^[^ ^4^, ^10^, ^12^, ^25^ ^]^ our hypothesis is centered on replicating this damage by exposing polymers to an aqueous environment rich in OH^•^ radicals. Successfully matching the resulting polymer damage, both morphologically and in particle deposition, outside the microscope would validate the recreation of radiolytic damage observed in the liquid cell. To further investigate this hypothesis, we initially employed a well‐established kinetic model^[^ ^10^, ^14^ ^]^ to theoretically predict the radiolytic environment in the liquid cell (**Figures** **3**a–c and Tables S2,  S3, Section S3.6, Supporting Information), and subsequently, an OH^•^ radical‐rich environment was created through the photolysis of hydrogen peroxide under UV irradiation (as illustrated in Figure 3d). Note that the whole kinetic model was solved by considering 17 species and 83 reactions (see details in Table S3, Section S3.6, Supporting Information).

Through modeling, we calculated the time‐dependent concentrations of four radiolysis products, namely e_h_^−^, OH^•^, H_2,_ and O_2_, under continuous TEM imaging conditions in aerated water. Additionally, we determined their steady‐state concentrations (C_ss_) as a function of dose rate (Figures 3a–c). The modeling results showed that the C_ss_ of radicals can be rapidly established within 1 millisecond (Figure 3b). More importantly, under relatively low electron flux conditions (0.05–2 e^−^ Å^−2^ s^−1^, corresponding to a dose rate range of ≈5 × 10^5^−2 × 10^7^ Gy s^−1^), the concentration of OH^•^ radicals was ≈30 times higher than that of e_h_^−^ (Figure 3c). This observation implies a preference for oxidative reactions in our model system, consistent with previous reports.^[^ ^10^, ^12^ ^]^

To replicate the oxidative environment in LP‐TEM, we employed the UV/H_2_O_2_ process, a known method for generating OH^•^ radicals (Section S3.7, Supporting Information).^[^ ^34^ ^]^ The goal of this model experiment is to approximate the key feature of LP‐TEM, i.e., hydroxyl‐rich radical environment, as hydroxyl radicals play a major role in causing radiolytic damage to polymers in LP‐TEM. Importantly, many of the reactions involving OH^•^ radicals in the UV/H₂O₂ process,^[^ ^35^ ^]^ as shown in Table S4 (Supporting Information), are highly similar to those occurring in LP‐TEM radiolysis. The reaction rate constants commonly used in kinetic models for the UV/H₂O₂ process are either identical or within the same order of magnitude as those used for water radiolysis in LP‐TEM.^[^ ^35^, ^36^ ^]^ This similarity supports the feasibility of replicating the main features of the LP‐TEM environment using the UV/H_2_O_2_ approach. In this experiment, a dispersion of PEG‐*b*‐PS stomatocytes (1.4 mg mL^−1^) was exposed to UV light (320–500 nm) in the presence of H_2_O_2_ under continuous N_2_ flow (Figure 3d). Note that water radiolysis induced by UV irradiation within the wavelength range used in our experiments can be neglected.^[^ ^37^ ^]^ Additionally, the steady‐state concentration of hydroxyl radicals created in the UV/H_2_O_2_ process can be experimentally quantified by using UV–vis spectroscopy, with salicylic acid (SA) as an OH^•^ probe (Scheme S2, Figure S24, Supporting Information).^[^ ^38^ ^]^ Preliminary results indicate that the OH^•^ concentration is on the order of 10⁻¹^2^ m in the presence of 6 mm H₂O₂ (see Section S3.7.2, Supporting Information for details).

Time‐resolved Cryo‐TEM (Figure 3e; Figure S25, Supporting Information) confirmed the gradual formation of small nanoparticles on the stomatocyte membrane surface upon UV irradiation, resembling the nanoparticles observed in LP‐TEM. Quantitative analysis of the size distribution shows the nanoparticles formed in UV/H₂O₂ experiments (5.04 ± 0.46 nm) are comparable to most of the initially formed nanoparticles in LP‐TEM, which range from 5.34 to 8.01 nm (Figure S26 and see Section S3.7.3, Supporting Information for further discussion). Extended UV irradiation resulted in the formation of visible agglomerates in the solution and the development of a foam‐like 3D insoluble network (Figure 3f). Subsequently, both the foam layer and the liquid layer were collected and characterized using dry‐TEM. Our dry‐TEM results showed that the foam layer was formed by aggregation of stomatocytes (Figure S27, Supporting Information) and, importantly, we found that the liquid layer contained similar intermediate fragmentation structures and dendritic aggregates as observed in the LP‐TEM results (Figures 3g,h; Figure S26, Supporting Information). The close match of observed features signifies the successful mimicking of similar radiolytic damage to the polymer using the UV/H_2_O_2_ approach.

Next, we investigated the changes to polymer dispersity and molecular weight upon irradiation using DLS, MALDI‐MS, and GPC, respectively. The DLS data (Figure 3i) obtained after 4 h of irradiation revealed a five‐fold increase in hydrodynamic diameter, attributed to membrane fragmentation, aggregation of stomatocytes, and nanoparticle deposition on the outer membrane as evidenced by dry‐TEM. MALDI‐MS results (Figure 3j) obtained from both foam and liquid layers indicated significant cleavage of the original PEG_2K_ chains of the PEG‐*b*‐PS block copolymer into much shorter PEG molecules. Notably, control experiments containing only 5 mg mL^−1^ of soluble PEG_2K_ solution under the same UV/H_2_O_2_ conditions did not exhibit distinguishable PEG peaks in the mass spectra (Figure S28, Supporting Information), suggesting complete degradation of PEG_2K_ polymers into low molecular weight compounds.^[^ ^34b^ ^]^ To elucidate changes in the molecular weight of PS chains, we conducted GPC analysis on samples from both the foam and liquid layers, which were freeze‐dried and dissolved in tetrahydrofuran (THF). Our GPC results (Figure 3k) revealed a shoulder peak forming after 4 h of UV exposure. This indicates an average twofold increase in PS molecular weight, implying the occurrence of cross‐linking within the PS chains.^[^ ^39^ ^]^

In summary, it can therefore be concluded that the formation of the stable foam results from a substantial decrease in stomatocyte solubility due to PEG chain scission, and that the nanoparticles deposited on the membrane consist predominantly of PEG‐*b*‐PS polymer fragments in both the liquid cell and UV/H_2_O_2_ experiments, where the PS chains have undergone cross‐linking. In future studies, we believe that kinetic modeling could be employed to further elucidate the kinetics of hydroxyl radical formation in the UV/H₂O₂ process.^[^ ^35a,c^ ^]^ This would allow for a better quantitative comparison of hydroxyl radical formation and its resulting polymer damage between the UV/H₂O₂ process and LP‐TEM radiolysis.

### 2.4 Scavenger‐Based Approaches to Mitigating Radiolytic Damage

**Figure 4:**
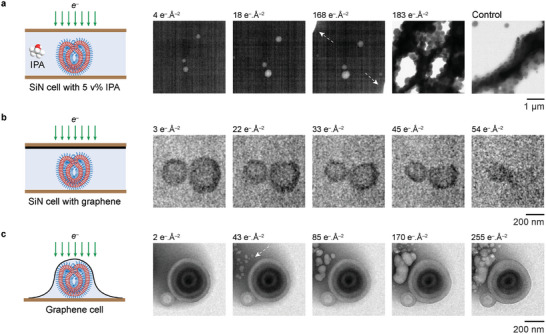
Approaches to mediating radiolytic damage to the polymer stomatocytes using hydroxyl radical scavengers. a) Using IPA (5 v%) in the SiN cell (see Movie S6, Supporting Information). The white arrows point out the liquid‐vapor interfaces. b) Introducing multilayer graphene on the top chip to protect the polymer sample (see Movie S7, Supporting Information). c) Encapsulating stomatocytes inside a graphene liquid cell (see Movie S8, Supporting Information). Electron flux: a,b) 0.60 e^−^ Å^−2^ s^−1^, c) 0.85 e^−^ Å^−2^ s^−1^.

After establishing the molecular process of degradation, we next focused on the use of hydroxyl radical scavengers to mitigate polymer damage in LP‐TEM. While the use of hydroxyl radical scavengers in various polymer systems has been extensively explored in the literature on radiation chemistry, there are not many studies on the scavenging efficacy of hydroxyl radical scavengers in LP‐TEM of polymers.^[^ ^4^, ^8^, ^12^, ^16^ ^]^ In particular, there has been a lack of comparative studies to assess which scavenger provides better protection in terms of the cumulative radiation dose the polymer system can withstand. Here we carried out a comparative analysis of two commonly used hydroxyl radical scavengers in LP‐TEM studies of polymers, namely IPA and graphene. These scavengers have two main differences: first, IPA induces changes in the initial chemical environment within the liquid cell, whereas graphene, being a solid, does not; and second, IPA converts the OH^•^ radicals into less reactive radicals for polymer damage, whereas graphene converts OH^•^ radicals into terminated species.^[^ ^4b^ ^]^ We fabricated two distinct SiN liquid cells: one containing 5 v% IPA in liquid water^[^ ^12b^ ^]^ (**Figure** **4**a and Movie S6, Supporting Information) and the other featuring multilayer graphene coated onto the top of a liquid cell chip (Figure 4b and Movie S7, Supporting Information). Detailed characterization of these graphene‐coated chips is available in Section S3.8 (Supporting Information) (Figures S29–S31, Supporting Information). For both liquid cells, the PEG‐*b*‐PS stomatocytes were initially loaded onto the bottom chip and allowed to dry before the cell was sealed and filled with water. Subsequently, the stomatocytes in both liquid cells were exposed to electron beam irradiation at the same electron flux of 0.60 e^−^ Å^−2^ s^−1^.

In the liquid cell with 5 v% IPA, we initially found only three stomatocytes with an air bubble trapped in the cavity. Remarkably, at a cumulative dose of 168 e^−^ Å^−2^, the liquid water was displaced from the viewing window area, giving rise to a 3D stomatocyte agglomerate (Figure 4a and Movie S6, Supporting Information). This agglomeration of particles suggests a substantial reduction in polymer solubility in liquid water, likely attributed to PEG chain scission in the bilayer membrane. This observation aligns with our findings from UV/H_2_O_2_ experiments, where decreased polymer solubility manifested as the formation of an insoluble 3D stomatocyte network (Figure 3). These observations emphasize the limited protective effect of IPA against polymer damage, especially for the hydrophilic chains.^[^ ^12b^ ^]^

In the liquid cell coated with multilayer graphene on the top chip with a cell thickness ranging from 550 to 850 nm, the most intriguing observation was the presence of significant stomatocyte shrinkage (Figure 4b; Figure S31, Movie S7, Supporting Information). These stomatocytes completely vanished within ≈120 s at a cumulative dose of 72 e^−^ Å^−2^, similar to their lifetime in the liquid cell without graphene. Notably, no nanoparticle deposition was observed in the graphene‐coated cell, even using an electron flux of 1.0 e^−^ Å^−2^ s^−1^ for 400 s. The scavenging mechanisms of graphene have been reported to involve either its reaction with hydroxyl radicals,^[^ ^16b^ ^]^ or its electrical conductivity and charge dissipation, which mitigate beam effects related to secondary electrons.^[^ ^40^ ^]^ When imaging polymer samples at low dose rates in the presence of O_2_ in water, the first mechanism‐scavenging of hydroxyl radicals is likely to be more dominant.^[^ ^10^, ^24^ ^]^ We therefore hypothesize that the effectiveness of graphene's protection is closely related to its distance from the sample. In essence, effective protection can only be achieved when the sample is close to graphene, thus, effectively reducing the concentration of hydroxyl radicals.

To validate this hypothesis, we imaged the same polymer stomatocytes encapsulated inside a graphene cell, where monolayer graphene enshrouded the outer vesicle membrane, and the vertical cell thickness was determined by the size of the trapped vesicles (ranging from 300 to 400 nm). We found that the entire outer membrane of the stomatocytes (≈25 nm thick), remained unscathed and pristine, with no significant volume shrinkage or nanoparticle formation (Figure 4c; Figure S32, Movie S8, Supporting Information). Notably, bubble formation events occurred near the stomatocyte, which could be attributed to various factors such as radiolysis‐induced hydrogen evolution, local pressure variation, and gas evolution as the byproducts of graphene oxidation.^[^ ^16b^ ^]^ This evidence strongly supports the notion that graphene's scavenging effect is indeed distance‐dependent. Within 25 nm, i.e., the membrane thickness, graphene effectively eliminates the OH^•^ radicals, preserving the bilayer vesicle membrane from radiolytic damage. Additionally, we observed slight degradation of the second inner membrane (located 40 nm from the outer membrane surface) within the stomatocyte, suggesting that 40 nm is likely beyond the effective scavenging distance of graphene under the dose rate conditions employed. This observation is likely influenced by the diffusion coefficient of hydroxyl radicals through a bilayer vesicle membrane and their reaction kinetics with graphene, which warrants further investigation. To explore this, we anticipate that using a pressure‐controlled approach to create a graphene‐coated SiN liquid cell with varying thicknesses could be a possible solution.^[^ ^28^ ^]^

## 3 Conclusions

In this work, we have investigated, mimicked, and sought to mitigate radiolytic damage to functional polymers in LP‐TEM. We thoroughly examine polymer damage under low dose conditions in vacuum, water vapor, and liquid water, both with and without hydroxyl radical scavengers. Our study quantitatively elucidates polymer damage behaviors across these environments and compares differences, particularly between vapor‐phase and liquid‐phase TEM experiments, which we attribute to differences in oxygen and hydroxyl radical concentrations. In addition, we extend our LP‐TEM analysis to crystalline organic systems, observing significant variability in damage pathways across individual crystals using liquid electron diffraction. Importantly, we demonstrate the OH^•^‐rich LP‐TEM environment can be approximated by employing the UV/H_2_O_2_ process, bridging the gap between radiation chemistry in the liquid cell and reactions in the bulk liquid. Finally, we evaluate the effectiveness of commonly used radical scavengers in protecting polymer samples during LP‐TEM studies, revealing that graphene's protective effectiveness is distance‐dependent. This study offers fundamental insights into the radiolysis of polymers in LP‐TEM and will contribute to a more rational design of future LP‐TEM experiments. This will open opportunities for real‐time observation of shape transitions, particle mobility, and drug encapsulation of polymer assemblies while minimizing artifacts caused by radiolytic damage.

Given the significant challenges in imaging beam‐sensitive polymers in (non‐)aqueous liquids using LP‐TEM, more systematic and rational experimental approaches are needed.^[^ ^11^ ^]^ To address this, we provide a roadmap of key steps for successfully studying polymers and organics in LP‐TEM, along with guidance on selecting the most suitable experimental setup for specific polymer systems (Section S3.8.3, Supporting Information). When designing LP‐TEM experiments, we recommend following the decision tree in ref. [4a] to evaluate the feasibility of LP‐TEM for addressing the specific research question in polymer science. Determining the dose budget and distributing it rationally are essential steps. Given the tradeoff between resolution and beam effects, it is essential to lower the spatial resolution to a level that still captures the features of interest in polymers, thereby helping to mitigate the challenge of radiolytic damage. Importantly, increasing the dose budget may require the use of radical scavengers or control of physical parameters, but it is crucial to ensure compatibility between the sample and scavengers (Table S5, Supporting Information). Utilizing such beam damage control strategies brings dynamic in situ imaging of soft matter processes within reach, offering a combination of spatial and temporal resolutions that are unattainable with other methods, such as super‐resolution microscopy and cryo‐TEM.

## Conflict of Interest

H.S., H.Z., and H.H.P.G. work for DENSsolutions B.V. The other authors declare no conflict of interest.

## Author Contributions

J.C.M.H., H.H.P.G., and H.F. supervised the study. H.W., H.F., and J.C.M.H. wrote the manuscript. H.W., H.S., H.F., and J.C.M.H. designed the research. H.W., H.S., R.A.J.F.O., S.L., J.S., J.W., R.R.M.J., X.L., Y.L., H.Z. and L.K.E.A.A. performed the experiments. H.W. developed and performed the LP‐TEM, GLC, and Cryo‐TEM experiments and quantitative analysis of the EM data. R.A.J.F.O. synthesized the polymer stomatocytes. H.H.P.G. supervised the Stream experiments. H.S. and H.H.P.G. designed graphene‐coated Stream chips. H.S., H.Z., and H.W. performed the Stream experiments. S.L., J.W., Y.L., and H.W. designed and performed the UV/H_2_O_2_ experiments. X.L. carried out the MALDI‐MS and GPC measurements. R.R.M.J. assisted in the LP‐TEM and cryo‐TEM experiments. H.W. carried out the radiolysis modeling. All authors contributed to the interpretation of the data and commented on the manuscript.

## Supporting information



Supporting Information

Supplemental Movie 1

Supplemental Movie 2

Supplemental Movie 3

Supplemental Movie 4

Supplemental Movie 5

Supplemental Movie 6

Supplemental Movie 7

Supplemental Movie 8

## Data Availability

The data that support the findings of this study are available from the corresponding author upon reasonable request.
