# Supplementary material for: Understanding, Mimicking, and Mitigating Radiolytic Damage to Polymers in Liquid Phase Transmission Electron Microscopy
Source: Adv Mater. 2024 Nov 16;36(52):2402987. doi: 10.1002/adma.202402987 (PMC11681318; doi:10.1002/adma.202402987)
Supplement: Supplementary file 1 — Supporting Information [file ADMA-36-2402987-s008.pdf]

ADVANCED MATERIALS

Supporting Information

for *Adv. Mater.*, DOI 10.1002/adma.202402987

Understanding, Mimicking, and Mitigating Radiolytic Damage to Polymers in Liquid Phase Transmission Electron Microscopy

Hanglong Wu, Hongyu Sun, Roy A. J. F. Oerlemans, Siyu Li, Jingxin Shao, Jianhong Wang, Rick R. M. Joosten, Xianwen Lou, Yingtong Luo, Hongkui Zheng, Loai K. E. A. Abdelmohsen, H. Hugo Pérez Garza, Jan C. M. van Hest* and Heiner Friedrich**

Supporting Information

Understanding, mimicking and mitigating radiolytic damage to polymers in liquid phase transmission electron microscopy

Hanglong Wu,[1,2]* Hongyu Sun,[3] Roy A. J. F. Oerlemans,[1] Siyu Li,[4] Jingxin Shao,[1] Jianhong Wang,[1] Rick R. M. Joosten[2,4], Xianwen Lou,[1] Yingtong Luo,[1] Hongkui Zheng,[3] Loai K. E. A. Abdelmohsen,[1] H. Hugo Pérez Garza,[3] Jan C. M. van Hest[1]*, Heiner Friedrich[2,4]*

[1] *Bio-Organic Chemistry, Institute for Complex Molecular Systems, Eindhoven University of Technology, P.O. Box 513, 5600 MB Eindhoven, the Netherlands*

[2] *Center for Multiscale Electron Microscopy, Department of Chemical Engineering, Eindhoven University of Technology, Eindhoven, The Netherlands*

[3] *DENSsolutions B.V., Informaticalaan 12, Delft, 2628 ZD, The Netherlands*

[4] *Laboratory of Physical Chemistry, Department of Chemical Engineering, Eindhoven University of Technology, Eindhoven, The Netherlands*

**Correspondence to:*

Hanglong Wu, hanglong@mit.edu

Heiner Friedrich, h.friedrich@tue.nl

Jan C. M. van Hest, j.c.m.v.hest@tue.nl

Contents

1. Materials 3
2. Instruments 3
3. Methods 4
3.1 Preparation of stomatocytes 4
3.2 Imaging stomatocytes in vacuum, H_2O vapor and H_2O + IPA vapor 8
3.3 Imaging polymer stomatocytes in liquid water 13
3.4 Imaging crystalline organic materials in liquid water 20
3.5 Movie analysis 22
3.6 Kinetic model 23
3.7 Photoirradiation experiments using UV/H_2O_2 26
3.8 Graphene-coated SiN cell and graphene liquid cell 32
4. Captions of Supplementary Movies 36
5. References 37

1. Materials

Organic solvents were obtained from Biosolve Chemicals. Methoxypoly(ethylene glycol) (2 kDa) was purchased from JenKem Technology USA. All other chemicals were acquired from either Sigma-Aldrich or TCI. Chemicals were used as received without further purification.

2. Instruments

Nuclear Magnetic Resonance Spectroscopy (NMR)

A 400 MHz Bruker Cryomagnet was used for proton nuclear magnetic resonance spectroscopy (^{1}H NMR). Residual non-deuterated solvent was used as reference for the chemical shifts (in ppm), relative to the internal standard tetramethylsilane.

Gel Permeation Chromatography (GPC)

Gel permeation chromatography (GPC) with THF as eluent (1.0 mL min^{-1}) was performed on a Shimadzu Prominence-I GPC system, equipped with PLgel 5 μm mixed-D column (Polymer Laboratories), RID-20A differential refractive index detector and PDA detector.

Dynamic Light Scattering (DLS)

The hydrodynamic size of the prepared stomatocytes was measured using a Malvern Zetasizer Nano ZSP equipped with a 633 nm laser and an avalanche photodiode detector. The hydrodynamic diameter of stomatocytes before and after UV exposure was measured using an Anton Paar Litesizer 500 equipment at 20 °C with a 628 nm laser.

Mass Spectrometry (MS)

Matrix-Assisted Laser Desorption/Ionization coupled to Time-of-Flight mass spectrometry (MALDI TOF MS) measurements were performed using an Autoflex Speed (Bruker, Bremen, Germany) instrument.

SciTEM

Ultra-low-volume liquid sample deposition on the liquid cell chip was achieved by using an automated piezoelectric-actuated liquid handling system, SciTEM (SCIENION AG, a CELLINK company, Germany).[1]

Dry transmission electron microscopy (Dry-TEM)

Dry-TEM was performed on a Tecnai 20 (type Sphera, Thermo Fisher Scientific) operated at 200 kV equipped with a LaB_6 filament and a 4k × 4k Ceta camera (Thermo Fisher Scientific).

Cryogenic TEM (Cryo-TEM)

Cryo-TEM was carried out on the TU/e cryoTITAN (Thermo Fisher Scientific) operated at 300 kV equipped with a field emission gun (FEG) at an extraction voltage of 3950 V, an autoloader, and a post-column Gatan energy filter (GIF, model 2002). Images were acquired using the Digital Micrograph software (Gatan) with a post-GIF 2K Gatan charge-coupled device (CCD) camera (model 794) at a cumulative dose of ~0.5 $e^{-}.Å^{-2}$ per image. Zero-loss energy filtering with a slit width of 20 eV was used to enhance contrast.

Cryo-TEM samples were prepared by pipetting 3 µL of sample solution onto a 200-mesh Quantifoil Cu grid with R 2/2 holey carbon films (Quantifoil Micro Tools GmbH, part of the SPT Life Sciences group). The TEM grid was plasma treated for 40 s using a Cressington 208 carbon coater prior to use. The grid was then blotted for 3 s (blotting force: -3) in a Vitrobot Mark IV (Thermo Fisher Scientific) at 100 % humidity, followed by vitrification in liquid ethane.

Liquid-phase TEM

LP-TEM experiments were mainly performed with two commercial *in-situ* liquid flow cell holders (type Ocean and type Stream, DENSsolutions B.V.) on a Tecnai 20 electron microscope (Thermo Fisher Scientific) operated at 200 kV equipped with a LaB_6 filament and a 4k × 4k Ceta camera. For the Ocean chips, silicon nitride (SiN) chips with a window dimension of 400 µm × 30 µm and a spacer thickness of 0 nm were used. For the Stream chips, SiN chips with a smaller window (typical window width = ~10 µm, spacer thickness = 200 nm) were used. The window membrane thickness of both Ocean and Stream chips is 50 nm. Note that to render the surface of the chips hydrophilic, all SiN chips were plasma treated with O_2 for 60 s using a Cressington 208 carbon coater prior to use. Details of the Ocean and Stream liquid cell configurations and the liquid cell assembly process can be found in our previous publications.[1b, 1c, 2] Movies were captured using the TEM Imaging & Analysis software (TIA, Thermo Fisher Scientific). See the main text and the Methods section below for details of the LP-TEM procedures for each experiment.

3. Methods

3.1 Preparation of stomatocytes

3.1.1 Synthesis of PEG_{45}-*b*-PS_{207}

The reaction scheme for the synthesis of PEG_{45}-*b*-PS_{207} was as follows (see Scheme S1):

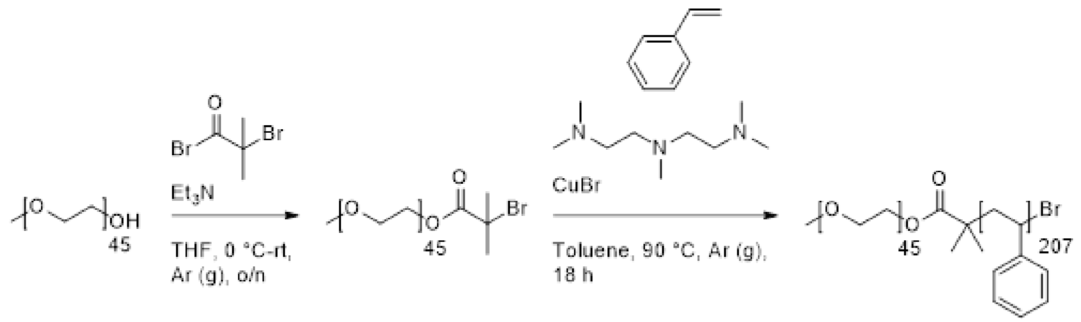

Scheme S1. Synthesis of PEG_{45}-*b*-PS_{207}.

α-Methoxy-poly(ethylene glycol)$_{45}$-ω-isobutyrylbromide, the atom transfer radical polymerization (ATRP) macro-initiator, was first synthesized according to the previously reported method with modifications.[3] Methoxypoly(ethylene glycol) (M_n=2.0 kDa) (3.00 g, 1.5 mmol, 1.0 eq), previously lyophilized from dioxane, was dissolved in 25 mL of anhydrous THF under Ar(g) atmosphere. Triethylamine (627 µL, 4.5 mmol, 3.0 eq) was added and the solution was cooled on ice. α-Bromoisobutyryl bromide (371 µL, 3.0 mmol, 2.0 eq) was added dropwise and the mixture was stirred overnight under Ar (g) atmosphere while warming to room temperature. The solids were then removed by filtration and the filtrate was concentrated under reduced pressure and taken up in 20 mL of toluene. Residual precipitates were removed by filtration and the solution was concentrated under reduced

pressure. The resulting oil was dissolved in approximately 6 mL of THF and precipitated in 250 mL of cold Et_2O. The precipitates were spun down (3046 rcf, 10 minutes) and the supernatant was removed. The pellet was resuspended in 50 mL of Et_2O and spun down again (3046 rcf, 10 minutes). The supernatant was removed and the pellet was lyophilized in dioxane to obtain α-methoxy-poly(ethylene glycol)$_{45}$-ω-isobutyrylbromide as a white solid (2.77 g, 1.3 mmol, 86%). The ^{1}H NMR result before purification is shown in Figure S1. ^{1}H NMR (400 MHz, Chloroform-*d*) δ = 4.40 – 4.29 (m, 2H), 3.64 (s, 180H), 3.38 (s, 3H), 1.94 (s, 6H).

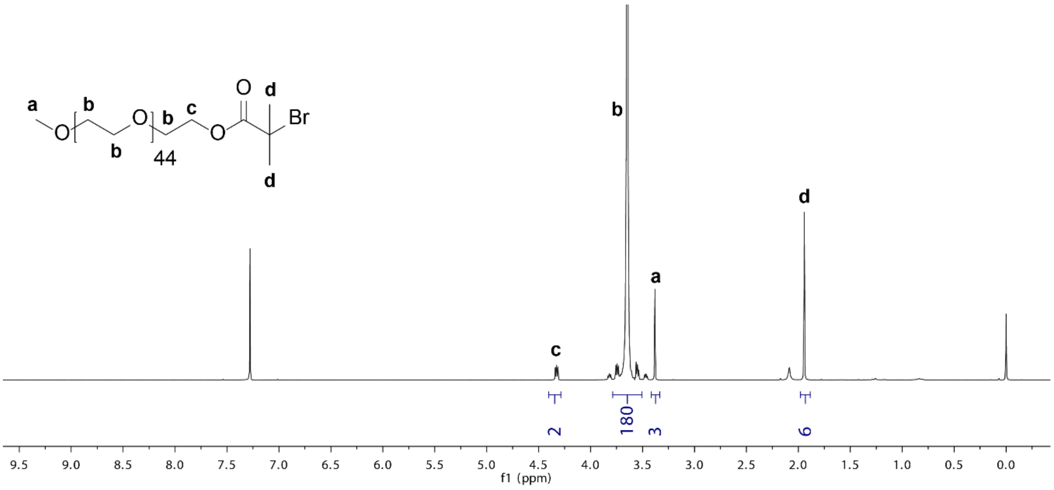

Figure S1. ^{1}H NMR spectrum of ATRP macro-initiator.

PEG$_{45}$-*b*-PS$_{207}$ was then synthesized according to the previously reported method with modifications.[3] Copper (I) bromide (46 mg, 0.32 mmol, 3.2 eq) was placed under Ar (g) atmosphere. Styrene (5.0 mL, 43.5 mmol, 435 eq) was added and the mixture was purged with Ar (g) for 15 minutes. *N,N,N',N",N"*-pentamethyldiethylenetriamine (67 µL, 0.32 mmol, 3.2 eq) was added and the mixture was purged with Ar (g) for 15 minutes. Toluene was first purged with Ar (g) for 15 min before dissolving α-methoxy-poly(ethylene glycol)$_{45}$-ω-isobutyrylbromide (215 mg, 0.10 mmol, 1.0 eq) in 1.5 mL of toluene, which was then added to the reaction mixture. The flask was transferred to a preheated oil bath of 90 °C and the mixture was stirred for 18 h under Ar (g) atmosphere. After the desired degree of polymerization was confirmed by ^{1}H NMR analysis, the mixture was cooled down to room temperature. Then it was diluted with dichloromethane (CH_2Cl_2) and filtered over neutral alumina. The filtrate was washed three times with 60 mM ethylenediaminetetraacetic acid (aq) and once with brine. The organic phase was dried over sodium sulfate (Na_2SO_4) and concentrated to approximately 10 mL. This was precipitated in cold methanol (MeOH). The precipitate was finally lyophilized from dioxane to yield a white powder, which was analyzed by ^{1}H NMR (Figure S2) and GPC (Đ= 1.13, Figure S3). ^{1}H NMR (400 MHz, Chloroform-*d*) δ = 7.22 – 6.21 (m, PS aromatic), 3.64 (s, PEG backbone), 3.38 (s, 3H, CH_3O), 2.29 – 0.74 (m, PS backbone + $C(CH_3)_2$).

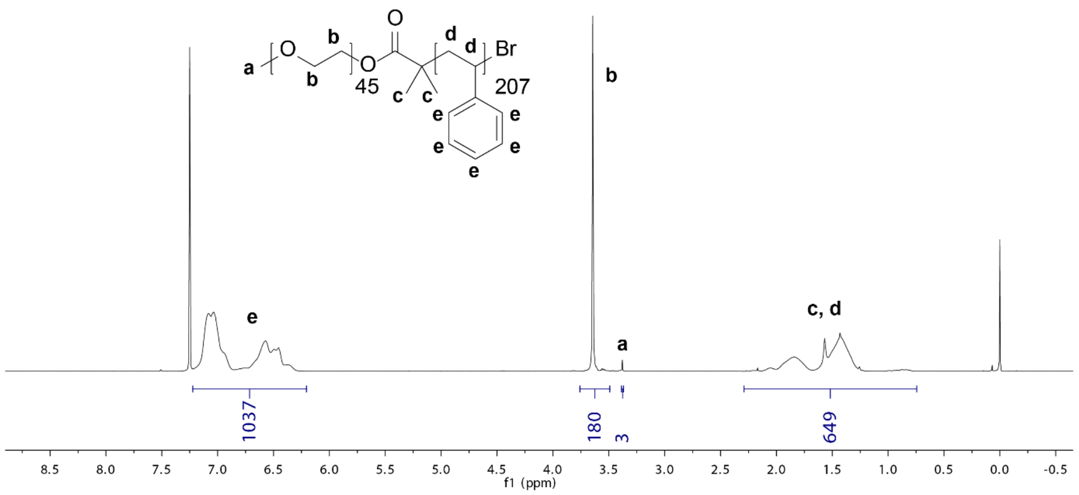

Figure S2. ^{1}H NMR spectrum of PEG$_{45}$-*b*-PS$_{207}$.

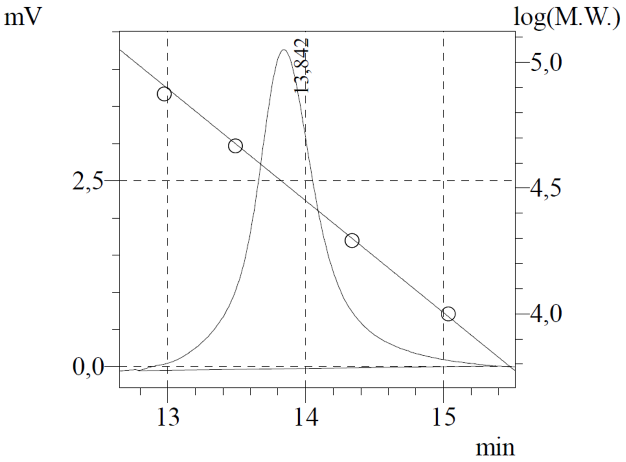

Figure S3. GPC trace of PEG$_{45}$-b-PS$_{207}$. Đ = M_w/M_n = 33389/29660 = 1.13.

3.1.2 Formation of PEG$_{45}$-*b*-PS$_{207}$ stomatocytes

PEG$_{45}$-*b*-PS$_{207}$ (20 mg) was dissolved in a 2.0 mL mixture of THF/dioxane (4:1 v/v), followed by the addition of 2.0 mL of MilliQ using a syringe pump at a rate of 1.0 mL/h with stirring (550 rpm). The resulting polymersome dispersion was transferred to a dialysis bag (molecular weight cut-off of 12-14 kDa (Spectra/Por)) and dialyzed against 1 L of 20 mM sodium chloride (aq). After one hour, the dialysis solution was replaced with 1 L of MilliQ and dialyzed for another 18 hours. The dialysis solution was again refreshed with 1 L of MilliQ and this was repeated after one hour. Finally, the stomatocyte dispersion was collected from the dialysis bag. An overview of the polymer stomatocyte formation process is shown in Figure S4. The resulting stomatocytes were then characterized by DLS (Figure S5), dry-TEM and cryo-TEM (Figure S6).

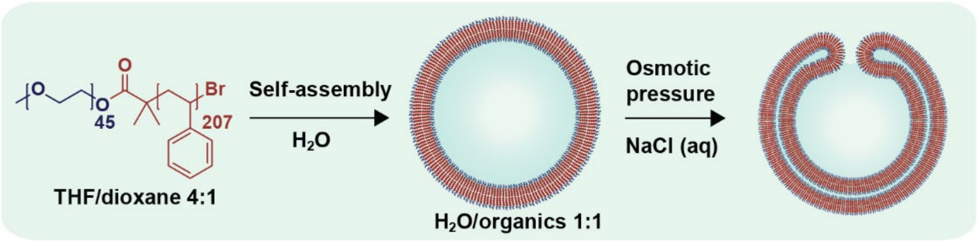

Figure S4. Schematic overview of PEG-*b*-PS self-assembly and subsequent shape transformation into stomatocytes.

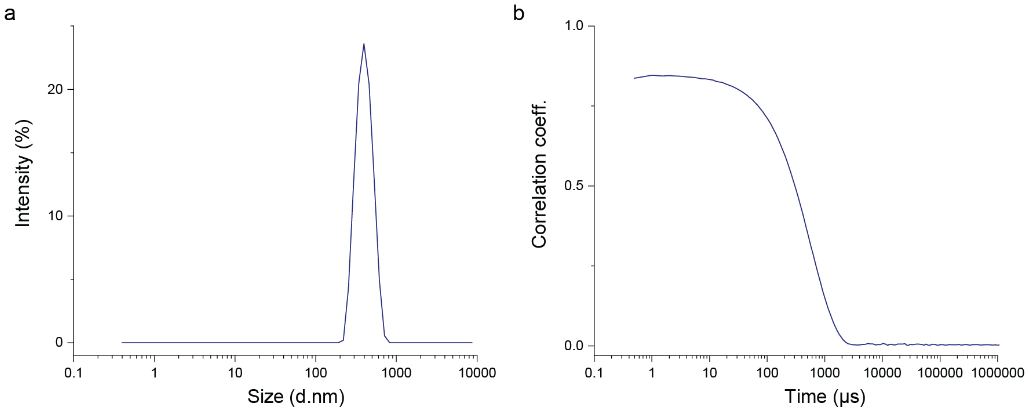

Figure S5. DLS analysis of PEG_{45}-*b*-PS_{207} stomatocytes with a Z-average of 392 nm and a PDI of 0.02. (a) intensity. (b) correlogram.

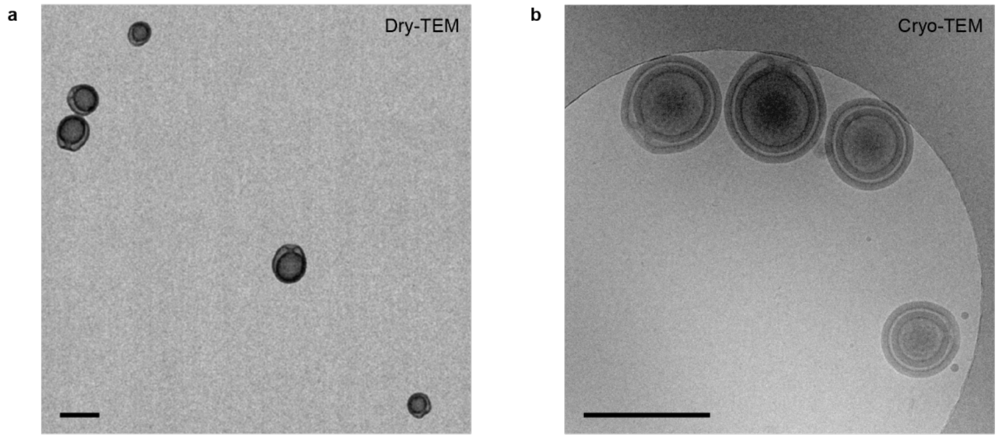

Figure S6. TEM characterization of stomatocytes. (a) dry-TEM and (b) cryo-TEM image of as-prepared PEG_{45}-*b*-PS_{207} stomatocytes. Scale bars: 500 nm.

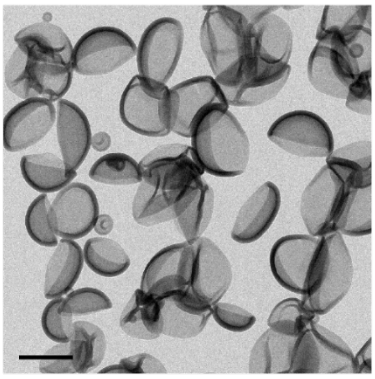

Figure S7. Dry-TEM image showing that initially spherical PEG-*b*-PS polymer vesicles can undergo significant structural deformation upon drying on the TEM grid. Scale bar: 500 nm.

3.2 Imaging stomatocytes in vacuum, H_2O vapor and H_2O + IPA vapor

3.2.1 Imaging stomatocytes in vacuum

A droplet containing 600 nL of the stomatocyte solution was first pipetted onto a SiN chip (type Ocean, DENSsolutions B.V.) and dried in an oven at 38 ºC for three hours. The SiN chip was then placed in a standard single-tilt TEM holder and exposed to electron irradiation with different electron fluxes (0.3-10 $e^{-}.Å^{-2}.s^{-1}$). The corresponding absorbed dose in Gy/s for 200 kV electrons is given in Table S1. The conversion from electron flux (ϕ, $e^{-}.Å^{-2}.s^{-1}$) to dose rate (ψ, Gy/s) can be calculated by[4]

$$\psi = (1 + \frac{t}{\lambda})S\frac{\phi}{e} \tag{1}$$

where t (nm) is the liquid layer thickness, λ (nm) is the inelastic mean free path of the liquid, S ($MeV.cm^{-2}.g^{-1}$) is the density-normalized stopping power in the medium, and *e* is the elementary charge. Values of λ and S at different electron energies are available in previous reports.[1b, 4b, 5] For our analysis, we use λ = 455 nm and S = 2.798 $MeV.cm^{-2}.g^{-1}$ at an electron energy of 200 keV. If the liquid layer thickness is significantly thinner than the electron's inelastic mean free path, i.e. $\lambda \ll t$, Eq. (1) can be simplified to:

$$\psi \approx S\frac{\phi}{e} \tag{2}$$

Our *in-situ* TEM results showed that even under extreme imaging conditions (10 $e^{-}.Å^{-2}.s^{-1}$ up to a cumulative dose of 5000 $e^{-}.Å^{-2}$), the stomatocyte experienced only a small area reduction (Figure S8), indicating that beam-induced damage to polymer stomatocytes in the dry state is negligible.

Table S1. Corresponding absorbed dose in Gy/s for 200 kV electrons at various electron fluxes

Electron flux $e^{-}.Å^{-2}.s^{-1}$	Dose rate ($\lambda \ll t$) Gy/s	Dose rate (400 nm) Gy/s	Dose rate (800 nm) Gy/s	Dose rate (1.5 μm) Gy/s
0.3	1.34×10^{6}	2.52×10^{6}	3.71×10^{6}	5.77×10^{6}
0.6	2.69×10^{6}	5.05×10^{6}	7.41×10^{6}	1.15×10^{7}
1.0	4.48×10^{6}	8.42×10^{6}	1.24×10^{7}	1.92×10^{7}
5.0	2.24×10^{7}	4.21×10^{7}	6.18×10^{7}	9.62×10^{7}
10.0	4.48×10^{7}	8.42×10^{7}	1.24×10^{8}	1.92×10^{8}

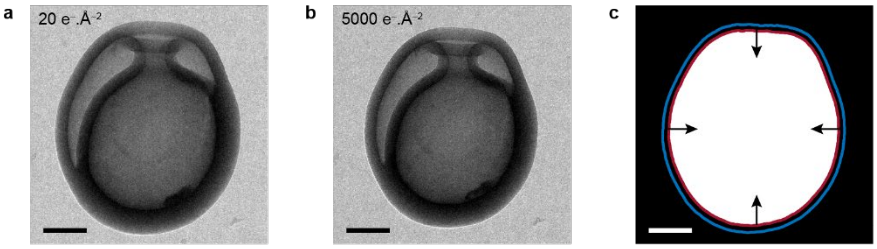

Figure S8. Assessment of electron damage to a stomatocyte under extreme imaging conditions in vacuum. (a-b) TEM images of a stomatocyte at a cumulative dose of (a) 20 $e^{-}.Å^{-2}$ and (b) 5000 $e^{-}.Å^{-2}$. Electron flux: 10 $e^{-}.Å^{-2}.s^{-1}$. (c) The contour changes of the stomatocyte before (blue) and after (red) exposure to electron irradiation at a cumulative dose of 5000 $e^{-}.Å^{-2}$, showing a slight area reduction. The arrows indicate the shrinkage of the stomatocyte. Scale bars: 100 nm.

3.2.2 Imaging stomatocyts in water vapor

Water vapor experiments with stomatocytes were primarily performed using the Ocean liquid flow cell holder (DENSsolutions B.V.). Two methods were developed to create the water vapor environment. (1) Using an ultra-low volume dispenser to assist with liquid handling and liquid cell assembly. This approach involved employing the sciTEM to deposit 20-50 nL of stomatocyte dispersion onto the bottom chip, after which the top chip was automatically loaded to make a sealed liquid cell. (2) Initially, 600 nL of the stomatocyte solution was first pipetted onto a SiN chip. After partial evaporation of water in the window area of the bottom chip (~1 min), the top chip was placed, assembling the liquid cell with two windows aligned in a cross fashion. This configuration yielded a viewing window area of 30 µm × 30 µm. Once the holder was inserted into the microscope column, MilliQ water was manually flowed into the liquid cell which appeared in the field of view at 550× magnification.

Using these methods, three different water vapor environments were created, as illustrated in Figure S9. (1) An environment where the stomatocytes were exposed to pure water vapor at 100% relative humidity (RH = 100%, Figure S9a). (2) A condition where the stomatocytes were surrounded by a thin layer of liquid water (Figure S9b). (3) An environment where the stomatocytes were at a liquid-vapor interface (Figure S9c). The variation in liquid water volume across three vapor environments leads to different hydroxyl radical concentrations near the stomatocytes, impacting their damage behaviors.

We noted that, in a water vapor environment, there should always be at least a thin water layer on the surface of hydrophilic PEG chains. Furthermore, water vapor may also condense on the silicon nitride membrane to form nanodroplets under electron irradiation as recently demonstrated by Woehl et al.[6] We have observed similar phenomena in our experiments (Figure S9). Consequently, it is challenging to completely exclude the effect of a thin wetting layer in water vapor studies on block copolymer assemblies. We have also summarized all three possible scenarios that polymer stomatocytes could be exposed to in the water vapor environment in the liquid cell in Figure S9. The effects of electron flux and cumulative dose on the dissolution process of polymer stomatocytes are shown in Figure S10-11. Additionally, we've quantified the dissolution kinetics of polymers by monitoring their intensity changes (Figure S14), revealing that the polymers trapped in an apparent water layer dissolve much faster than those without an apparent water layer around them. We speculate that this difference is related to the hydroxyl radical concentration and the tunneling-like effect of oxygen gas molecules occurring at the solid–liquid–gas interface.[7]

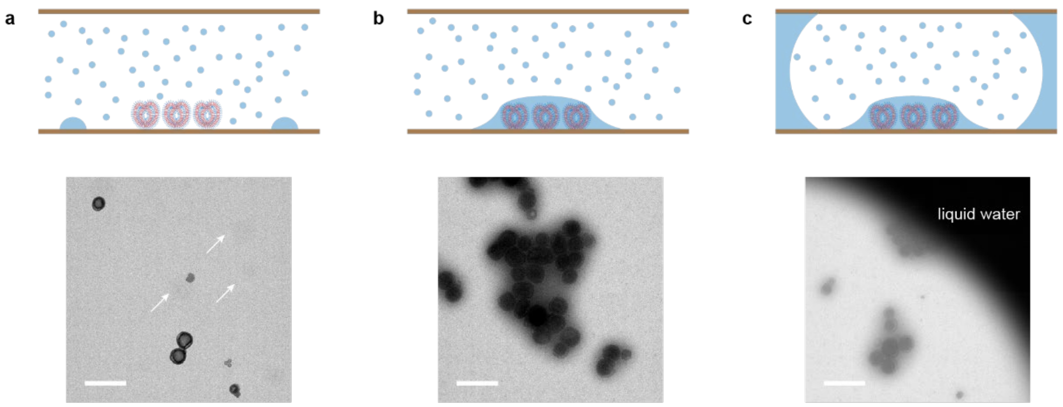

Figure S9. Exposure of stomatocytes to three different H_2O vapor environments within the SiN liquid cell. (a-c) Schematics and corresponding TEM image showing several stomatocytes exposed to (a) pure water vapor, (b) surrounded by a thin layer of liquid water and (c) trapped at a liquid-vapor interface close to a meniscus. The white arrows indicate some liquid water islands resulting from water vapor condensation. Scale bars: 1 μm.

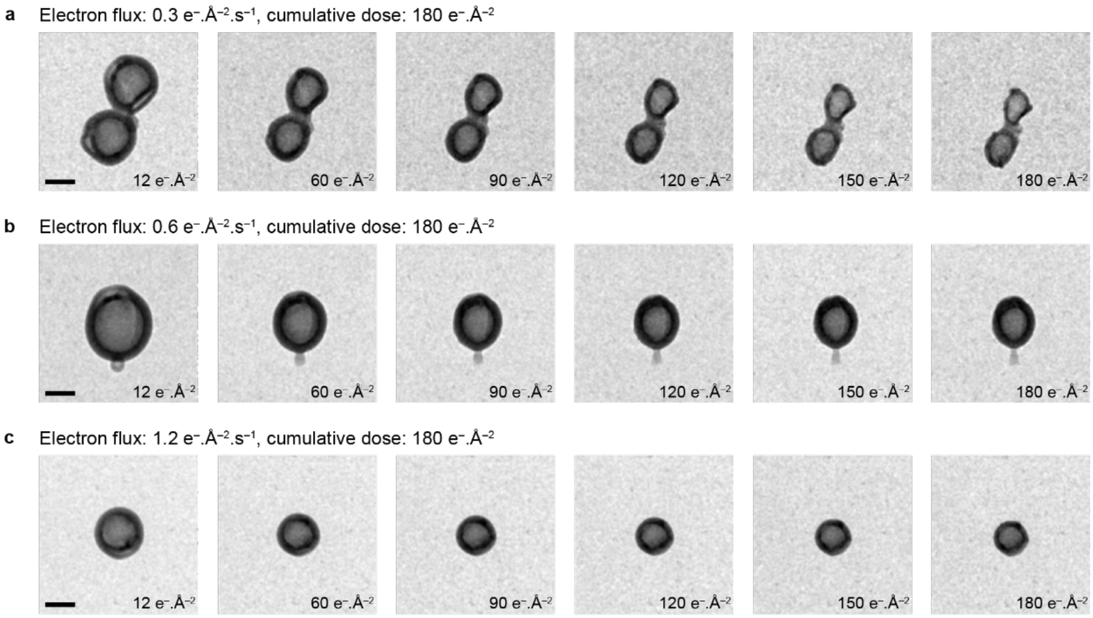

Figure S10. TEM image sequences showing the dissolution process of polymer stomatocytes in pure H_2O vapor at different electron flux conditions. Note that there was no visible liquid water around the stomatocytes. (a) 0.3 e$^-$.Å^{-2}.s^{-1}**.** (b) 0.6 e$^-$.Å^{-2}.s^{-1}. (c) 1.2 e$^-$.Å^{-2}.s^{-1}. Scale bars: 200 nm. Our results indicate that the dissolution kinetics of polymer stomatocytes are not mainly influenced by the electron flux, since increasing the electron flux by a factor of 4 has negligible effects on the polymer dissolution process.

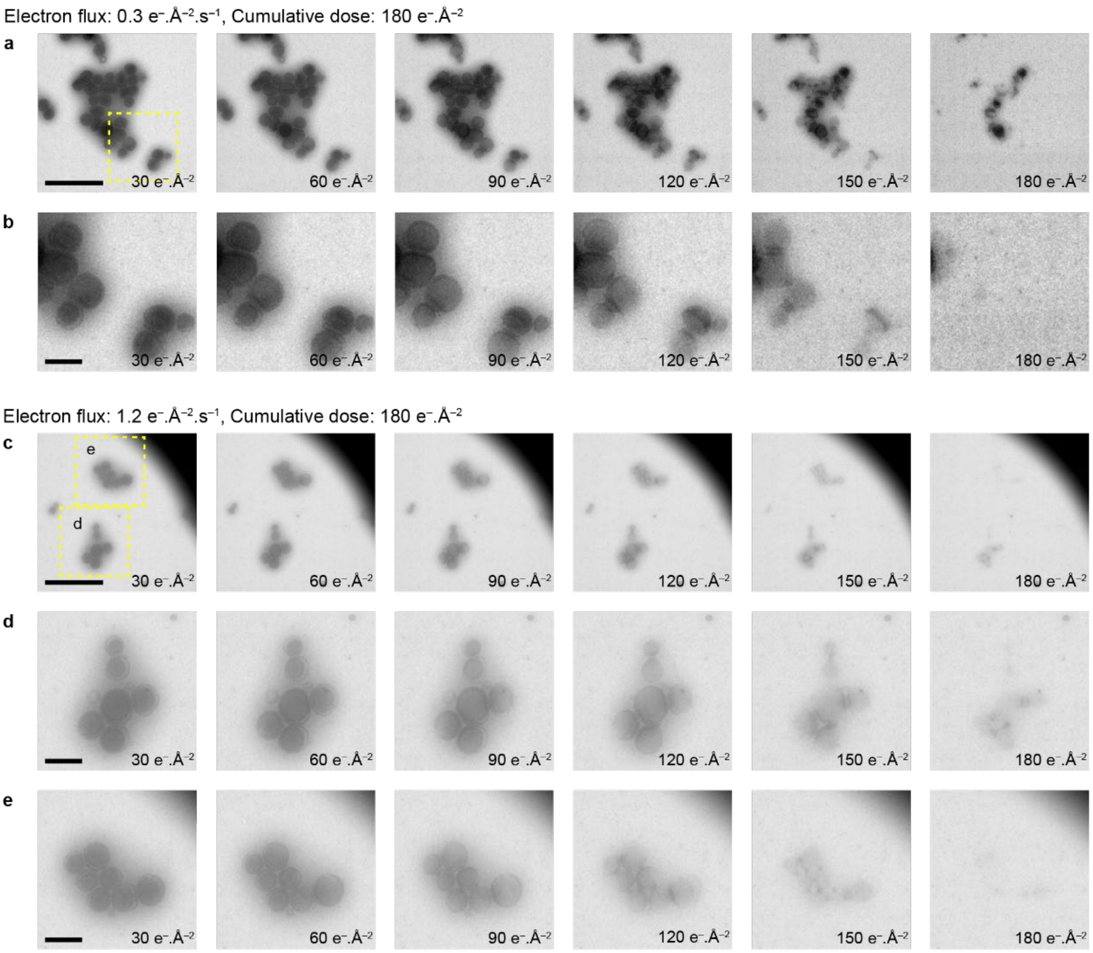

Figure S11. TEM image sequences showing the dissolution process of polymer stomatocytes trapped at the liquid-vapor interface. (a) TEM image sequences showing the dissolution process of several stomatocytes surrounded by a thin layer of liquid water at an electron flux of 0.3 $e^{-}.Å^{-2}.s^{-1}$. (b) Enlarged TEM image sequences from the area highlighted by the yellow box in (a). (c) TEM image sequences showing the dissolution process of polymer stomatocytes trapped at the liquid-vapor interface at an electron flux of 1.2 $e^{-}.Å^{-2}.s^{-1}$. (d-e) TEM image sequences showing the areas highlighted by the yellow boxes in (c) where the stomatocytes in area (e) are closer to a concave meniscus compared to those in area (d). Scale bars: (a, c) 2 μm. (b, d-e): 500 nm.

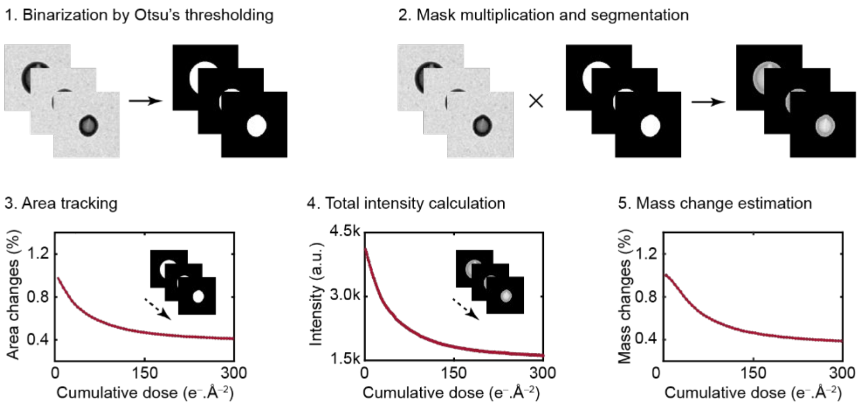

Figure S12. Image processing procedures for tracking the projected area and estimating mass changes in polymer stomatocytes under electron irradiation. Note that the image stack has been aligned to correct for sample drift prior to image binarization. See SI section 3.4 for further details.

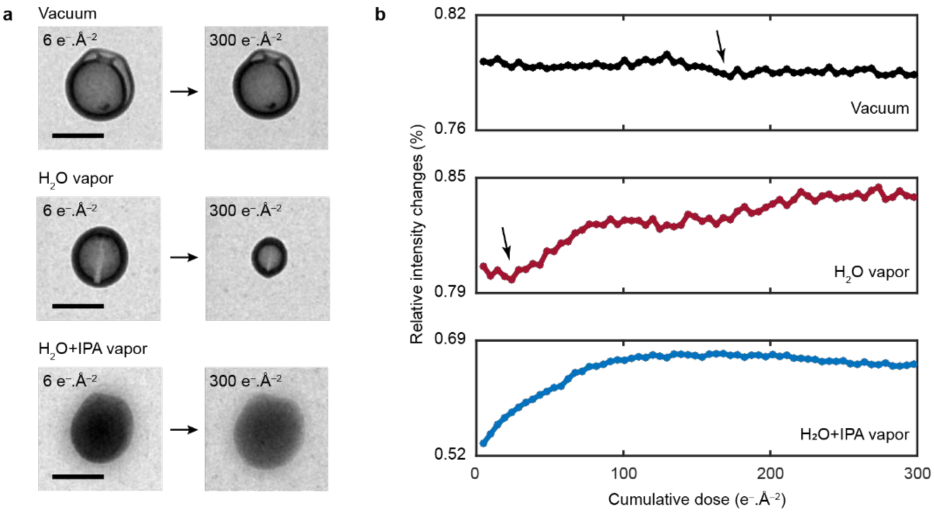

Figure S13. Radiolytic damage to polymer stomatocytes in vacuum, H_2O vapor and H_2O + IPA vapor. (a) TEM images showing the morphological changes of polymer stomatocytes at a cumulative dose of 300 e$^-$.Å^{-2}. (b) Averaged relative intensity of the stomatocyte *versus* cumulative dose under three different environments. Each data point is averaged over 25 pixels at the center of the stomatocyte. The arrows in (b) indicate a slight decrease in TEM intensity, suggesting the presence of a small degree of polymer densification. Electron flux: 0.6 e$^-$.Å^{-2}.s^{-1}. Scale bars: 200 nm.

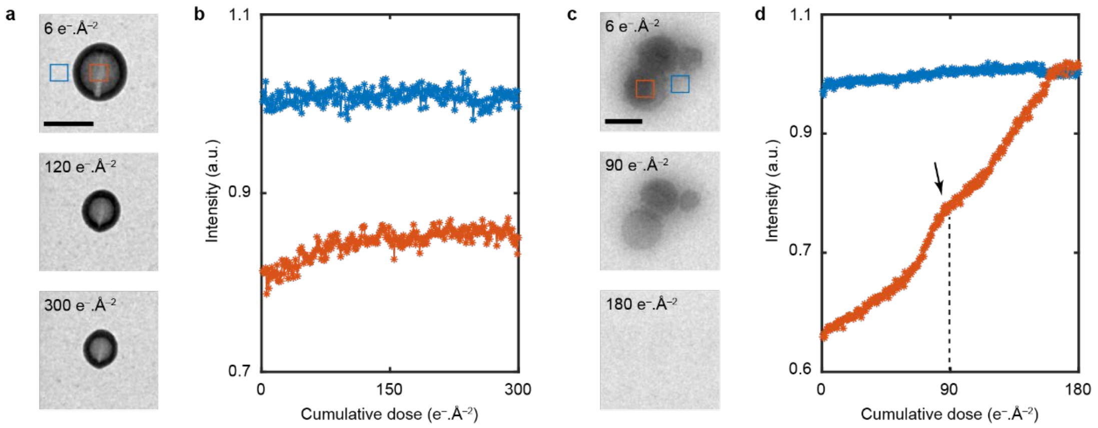

Figure S14. Monitoring the intensity changes near and on the imaged stomatocytes with cumulative dose. (a-b) TEM images of a stomatocyte experiencing area shrinkage in pure H_2O vapor at different cumulative doses (a) and corresponding intensity *versus* cumulative dose plots (b) derived from the areas highlighted in (a). (c-d) TEM images showing the dissolution process of several stomatocytes surrounded by a thin layer of liquid water in H_2O vapor (c) and corresponding intensity *versus* cumulative dose plots (d) derived from the areas highlighted in (c). Each data point in (b) and (d) is averaged over 25 pixels. The arrow in (d) indicates that at a cumulative dose of 90 e$^-$.Å^{-2} stomatocyte flattening occurred, resulting in a sudden increase in the average intensity in the plot. Our results indicate that there was no detectable material deposition on the SiN membrane during polymer dissolution. Scale bars: 200 nm.

3.2.3 Imaging stomatocyts in H_2O + IPA vapor

The procedures for introducing a mixture of H_2O and IPA vapor into the liquid cell are similar to those described in 3.2.2 with two key distinctions: (1) The stomatocyte solution contains 5 v% IPA; (2) Instead of using pure MilliQ water, a solution containing 5 v% IPA is flowed into the liquid cell. Importantly, we have established that the original morphology of the stomatocyte remains unchanged after a two-

hour exposure to 20 wt% IPA in a vial (Figure S15). Consequently, we can conclude that any morphological changes observed in the stomatocytes within the H_2O + IPA vapor under electron irradiation are caused by the electron beam.

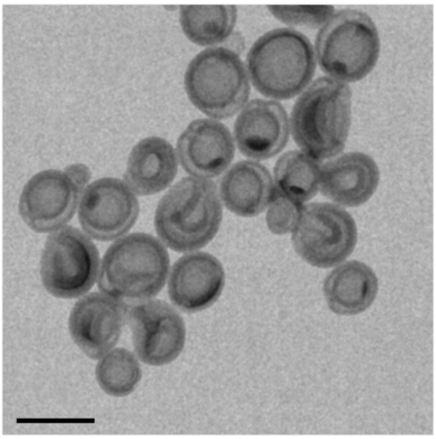

Figure S15. Dry-TEM image showing that the addition of IPA has a negligible effect on the original stomatocyte morphology. The TEM sample was prepared two hours after the addition of IPA to the stomatocyte solution (1 mg/mL). IPA concentration: 20 wt%. Scale bar: 500 nm.

3.3 Imaging polymer stomatocytes in liquid water

3.3.1 SiN liquid cell with a thick water layer

An aliquot (600 nL) of stomatocyte solution (1 mg/ml) was sandwiched between two SiN chips (type Ocean, DENSsolutions B.V.) by drop casting, resulting in the formation of a rather thick liquid cell in all 10 attempts (> 1 μm). LP-TEM imaging was performed using the Ocean liquid flow cell holder. A typical liquid cell prepared using this method is shown in Figure S16a, where the edges of the viewing window are blurred due to electron scattering from the thick layer of liquid water. Due to the large thickness, the stomatocytes were not visible in the first few seconds at an electron flux of 0.6 $e^{-}.Å^{-2}.s^{-1}$, but with increasing cumulative dose, several stomatocytes appeared with increasing contrast and membrane thickness over time (Figure S16-17). It should be noted that direct loading of stomatocytes into the liquid cell by drop casting often leads to nanoparticle accumulation around the edges of the window, with very few nanoparticles in the viewing window.

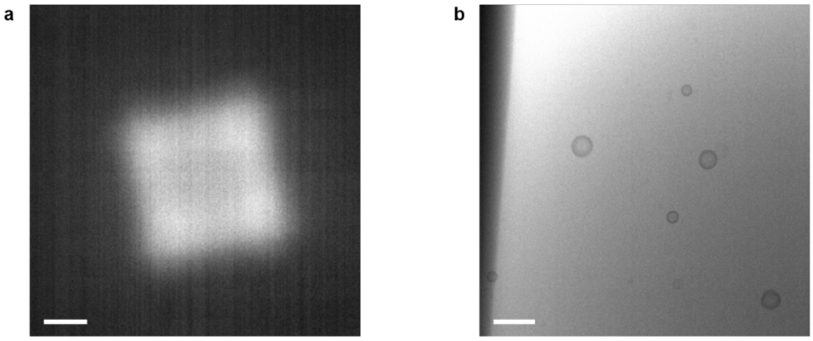

Figure S16. LP-TEM imaging of stomatocytes through a thick layer of liquid water. (a) LP-TEM image showing the entire viewing window of the liquid cell. (b) LP-TEM image of several stomatocytes with an exposure time of 8 s. Scale bars: (a) 10 μm. (b) 1 μm.

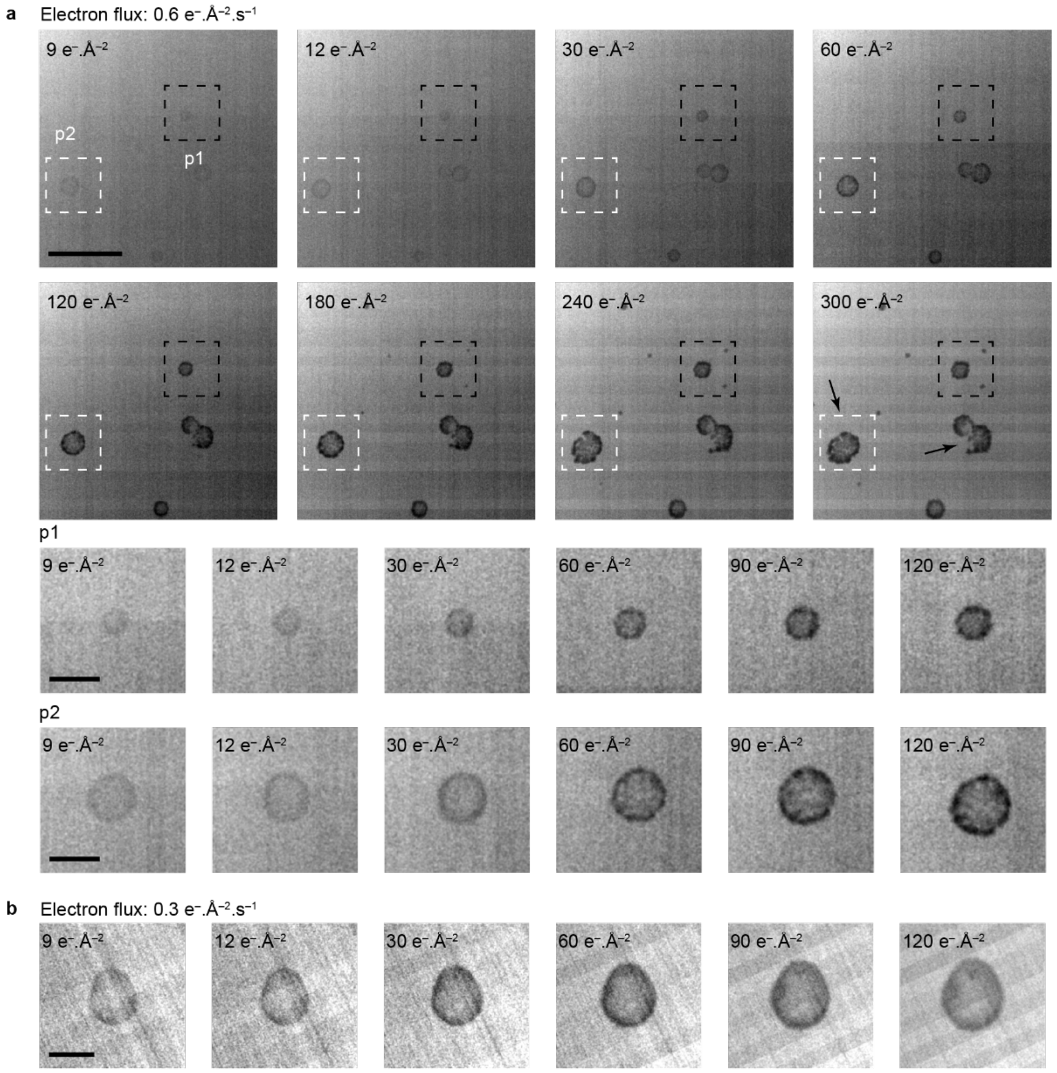

Figure S17. Monitoring radiolytic damage to stomatocytes in a thick layer of liquid water as a function of cumulative dose. LP-TEM results reveal that contrast and membrane thickness increase with cumulative dose at varying electron fluxes. Electron flux: (a) 0.6 e$^-$.Å^{-2}.s^{-1}. (b) 0.3 e$^-$.Å^{-2}.s^{-1}. At a cumulative dose of ~240 e$^-$.Å^{-2}, some stomatocytes in (a) were completely damaged, transforming into fragments as indicated by the arrows. The dashed black boxes highlight the evolution of stomatocyte p1 under the electron irradiation, as shown in Figure 2 of the main text. The white dashed boxes highlight the beam-induced morphological changes of another stonatocyte (p2). Zoom-in single-frame sequences of p1 and p2 are also shown in (a). Notably, at a cumulative dose of ~160 e$^-$.Å^{-2}, the area of p1 and p2 increased by ~120% and ~80%, respectively, compared to their original sizes. In contrast, the stomatocyte exposed to an electron flux of 0.3 e$^-$.Å^{-2}.s^{-1} increased by only 60% at the same cumulative dose. Scale bars: (a) 2 µm, (b) 500 nm.

3.3.2 SiN liquid cell with a thin water layer

To prepare a SiN cell with a thinner liquid water layer, we used SiN chips with a smaller viewing window (typical window width = ~10 μm). In addition, an automated piezoelectric-actuated ultra-low volume dispenser, the sciTEM, was employed to assist sample preparation. Typically, 5 droplets of stomatocyte dispersion (~300 pL/droplet, ~1.5 nL in total) were deposited onto the center of the viewing window and dried in the ambient environment. TEM analysis showed that no large particle accumulation was present (Figure S18a). After assembling the liquid cell, ultrapure MilliQ water was flowed into the liquid cell by a pressure pump at a pressure of 300 mbar and the liquid thickness was estimated using our earlier published method (Figure S18b-d).[1b] Figure S18d shows that a liquid water thickness of ~600 nm was achieved in the corner of the viewing window, allowing the investigation of early-stage stomatocyte damage induced by the electron beam (Figure S18e-g).

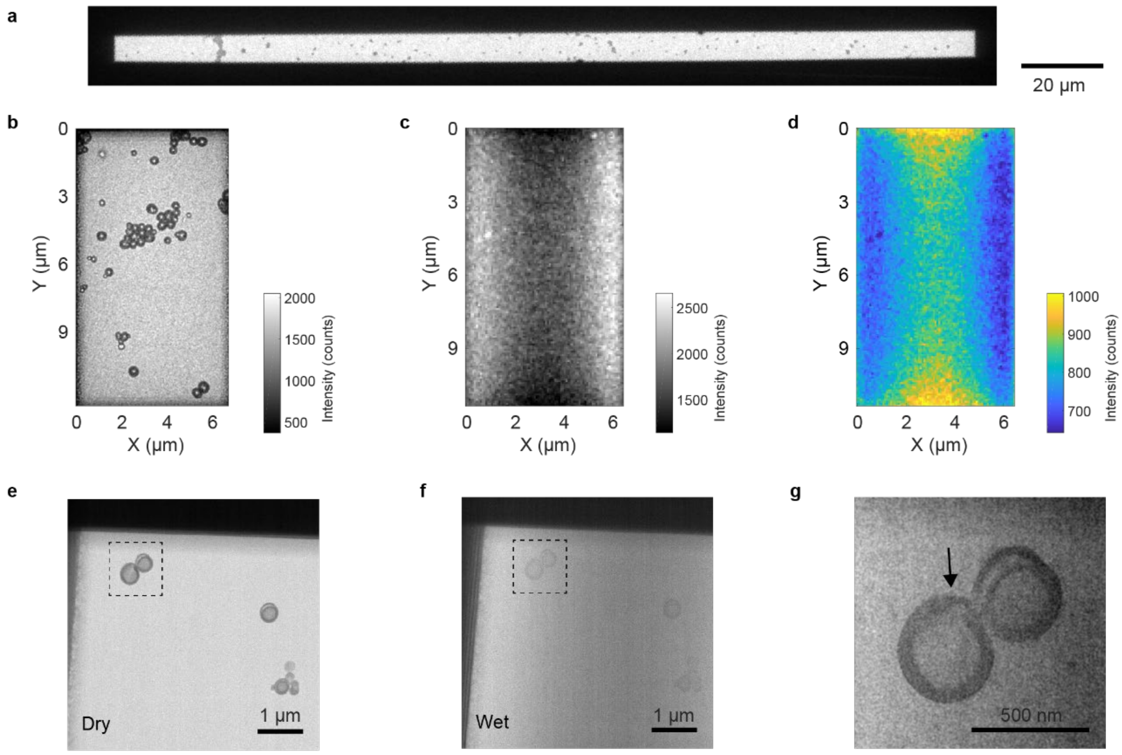

Figure S18. **LP-TEM of stomatocytes through a thin water layer.** (a) Overview of the spatial distribution of stomatocytes on the bottom chip after loading ~1.5 nL aliquot (1 mg/mL) using the sciTEM dispenser. (b-c) Overview of the liquid cell before (b) and after (c) filling with water. (d) Liquid thickness map of the cell. (e-f) Bright-field TEM images of stomatocyte nanoparticles before (e) and after (f) filling the cell with water. (g) Enlarged image (30-frame average) of two stomatocytes highlighted by the dashed box in (f), where the black arrow points to the neck of a stomatocyte.

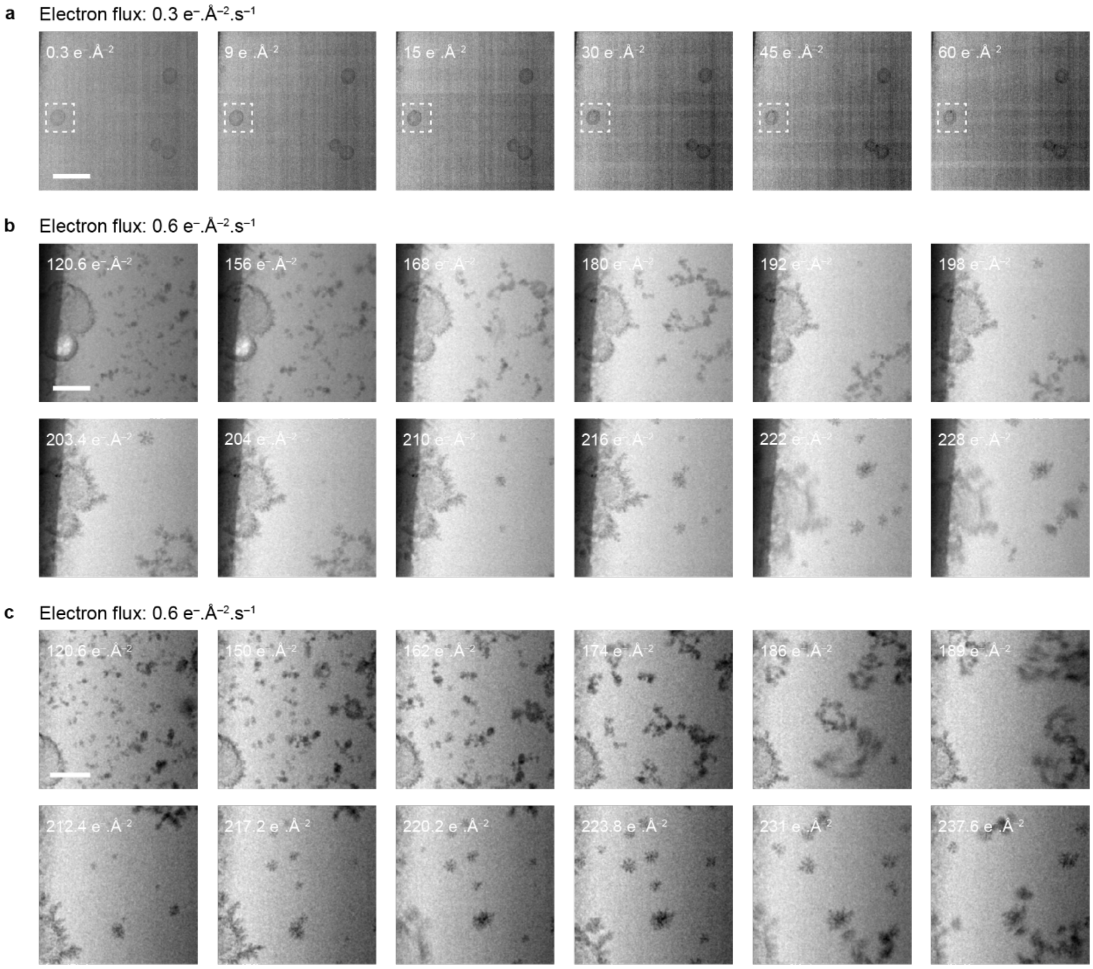

Figure S19. **Radiolytic damage to polymer stomatocytes in a thin layer of liquid water.** (a) LP-TEM image sequence depicting initial damage stages of polymer stomatocytes under electron irradiation. White boxes highlight the stomatocyte shown in Figure 2h of the main text. (b) LP-TEM image sequence showing the attachment of preformed dendritic aggregates to the damaged stomatocyte, the release of a trapped air bubble from the cavity of the stomatocyte, and the simultaneous dendritic nanoparticle formation and aggregation during electron irradiation. This process is partially depicted in Figure 2i of the main text. (c) LP-TEM images capturing the formation and aggregation processes of dendritic nanoparticles. Selected frames of this process are shown in Figure 2j in the main text. Scale bar: (a) 1 µm. (b-c) 500 nm.

3.3.3 Discussion on the confinement effects in the liquid cell

When studying polymers using LP-TEM, it is crucial to consider the nanoscale confinement imposed by the liquid cell. The strong interactions between the polymer samples and the enclosing SiN membranes as well as the limited liquid volume (in the pL to nL range), can alter many intrinsic characteristics of polymer systems, including their morphologies, structures, motion, formation, phase changes and stimuli-response behaviors.[2, 8]

3.3.3.1 Motion

In bulk solution, particle movement typically follows normal Brownian motion. However, in LP-TEM, particles often display complex anomalous diffusive motion due to heterogeneities, entrapment, crowding, and confinement imposed by the membrane and the local liquid environment.[8a-c] The diffusive motion of nanoparticles in most LP-TEM experiments has been found to be significantly slower than in bulk liquid, with diffusion coefficients several orders of magnitude smaller than those observed in bulk solution.[8d, 9] This slower movement has been attributed to inhomogeneous charging of the SiN membrane or the presence of a thin, highly viscous ordered liquid at the membrane interface.[8d] Specifically for polymer systems, constrained motion has been observed due to their continuous interaction with the membrane. For example, block copolymer micelles with a radius of 20-60 nm have been shown to display sub-diffusive fractional Brownian motion in the liquid cell, as revealed by the anomalous diffusion object motion analysis.[8a, 8c] To observe unhindered, bulk level diffusion, it has been suggested that using low electron flux conditions and increasing the liquid layer thickness could be beneficial.[8d]

3.3.3.2 Reaction kinetics

The reaction processes of polymers, such as self-assembly of polymers, within the liquid cell are highly dependent on the supply and depletion of reactants, which are determined by the liquid layer thickness (i.e. the separation between liquid cell membranes) and the flow configuration (e.g. bypass flow cell, direct flow cell and diffusion flow cell).[1c, 8b, 10] In bypass flow cells, when the gap between the two microchips becomes too narrow (< 500 nm), mass transport within the liquid cell can be significantly suppressed due to increased flow resistance. For example, the chemical etching of zeolites in a bypass cell was found to be more than six times slower than in a vial.[1a] As a result, the formation, phase changes and stimuli-responsive behaviors of polymer, which are highly dependent on the changes in local chemical environment, may take much longer in such cells compared to bulk solutions. In contrast, reaction kinetics in direct flow cells and diffusion cells can be much faster than in bypass cells, due to the more efficient liquid exchange between the windows. As demonstrated in a previous study, the etching kinetics of silica in an alkaline medium observed using LP-TEM was comparable to those obtained from in flask experiments, despite the more than ten orders of magnitude difference in liquid volume.[1c] Furthermore, regardless of the flow cell type, liquid exchange at the window membrane interface can be exceptionally slow compared to the center of the liquid cell due to the bulging of the window membrane.[11] As a result, local depletion of regents can frequently occur at these membrane interfaces.

Importantly, the strong interaction with the window interface, local depletion of polymer concentration, and varying solvent mixing conditions can shift the growth and phase transformation pathways of

polymers from thermodynamically controlled to kinetically trapped pathways within the liquid cell, resulting in morphologies significantly different from those observed in bulk solution.[2, 12] For amphiphilic systems, the hydrophilic surface of the SiN membrane surface can strongly influence the nucleation and growth of assemblies, thus affecting their final structural properties. In most LP-TEM experiments on polymers, polymer assemblies are often observed to form and adhere to the viewing window surface, allowing for more quantitative analysis of individual polymer due to a reduced motion blur.[2, 8a]

3.3.3.3 Morphology and structure

The morphology and structure of polymers can be influenced by spatial constraint in the liquid-cell environment. When studying pre-formed polymer assemblies in LP-TEM, if the size of the sample is larger than or similar to the spacer thickness, it is likely to be deformed during liquid cell assembly, a situation that should be avoided. But for smaller nano-object, the effects of confined environment on their size are generally negligible.[8c] For growing polymer assemblies *in-situ* in the liquid cell, the resulting structures can either resemble or differ significantly from those formed in bulk solution, depending on the thermodynamic and kinetic factors influencing the reaction process, as described in Section 3.3.3.2. Evidence has shown that polymer vesicles formed in the liquid cell can exhibit a similar spherical shape and membrane thickness to those formed in the laboratory, though their size is slightly smaller in the liquid cell.[2] Conversely, this is not the case in peptide systems. During peptide assembly, spherical nanoparticles have been found to dominate in liquid cell experiments, whereas fibers were mainly formed in vial experiments probably due to confinement effects on their reaction kinetics.[12]

In summary, to fully understand the confinement effects on polymer systems in LP-TEM, it is highly recommended to perform a variety of control experiments. This includes varying the thickness of the liquid layer, employing different flow configurations and beam-off conditions, and importantly always correlating LP-TEM results with those obtained from in-flask experiments. Conducting quantitative motion analysis of polymer nanoparticles can provide key insight into their motion mechanisms.[8a, 8b, 8d] Additionally, combining LP-TEM movie data with modelling may help to determine whether the formation process of the polymer assemblies under study is governed by thermodynamic or kinetic control.[2]

3.3.4 Discussion on the effects of electron dose on spatial resolution

The spatial resolution of LP-TEM is closely tied to the electron dose, and this relationship has been quantitatively detailed by de Jonge *et al.*[13] It has been shown that the spatial resolution for nano-objects in LP-TEM, d, scales with $D^{-1/4}$,[13] where D represents the electron dose. The $D^{-1/4}$ relation indicates when imaging beam-sensitive systems in LP-TEM, we can significantly reduce the required dose and only cause a relatively small decrease in spatial resolution.[13b] This is particularly useful for studying polymer assemblies (*e.g.* micelles, vesicles and cylinders) in LP-TEM, since the structural features of interest often fall within the range of a few to tens of nanometers. Given the tradeoff between resolution and beam effects, it is essential to lower the resolution to a level that still resolves the features of interest in polymers, which helps mitigate the challenge of radiolytic damage. For example, we previously demonstrated that from relatively low-resolution LP-TEM data (pixel size ≈ 7 nm/pixel), we were able to monitor the evolution of size and membrane thickness in polymer vesicles and gain new insights into their self-assembly mechanisms.[2]

In our thin water layer liquid cell experiments (Figure 2h in the main text and Figure S26), conducted at a cumulative dose of 1.5 $e^{-}.Å^{-2}$, with an electron flux of 0.30 $e^{-}.Å^{-2}.s^{-1}$, the spatial resolution was estimated to be around 5 nm using the 25–75% edge width approach over a nanoparticle formed on the stomatocyte.[11, 14] Additionally, we also performed liquid electron diffraction on a crystalline semi-organic system, zeolitic imidazolate framework-8 (ZIF-8) nanoparticles, directly in the SiN cell with a thinner liquid water layer, using an electron flux of 0.65 $e^{-}.Å^{-2}.s^{-1}$. Reproducible {110}a Bragg spots (D-spacing: 1.20 nm) and occasional {200} Bragg spots (D-spacing: 0.85 nm) were observed (Figure S22), indicating that information transfer to 1 nm or even sub-nm levels can be achieved under these low-dose conditions in our SiN cell. However, to more accurately evaluate the achievable spatial resolution, Fast Fourier Transform (FFT) analysis is recommended for extracting information from high-resolution real-space images. This approach, however, requires a much higher electron dose, likely exceeding the dose tolerance of ZIF-8 in liquid water.

3.3.5 Discussion on the effects of dose rate (absorbed power)

Beam effects, particularly radiolytic damage in LP-TEM, have been recognized as dose-rate (or absorbed power) dependent. This dependency arises because water radiolysis and the associated radical formation are dependent on the dose rate, which dictates the steady-state radical concentrations.[4a, 15] As a result, varying the dose rate by several orders of magnitude can lead to a range of different phenomena. For instance, as noted by Schneider et al, the etching and growth of metal nanoparticles could be seen at different dose rates due to the change in the ratio of reducing to oxidizing radical concentrations.[4a] Similarly, we have also observed that zeolite nanocrystals in alkaline solution exhibit distinct different radiolytic damage pathways depending on the used dose rates.[16]

Dose rate effects in cryo-TEM are fundamentally different from those in LP-TEM. In cryo-TEM, the formation and diffusion of radicals are significantly suppressed, leading to more localized radiolytic damage. Notably, our previous cryo-TEM work on organic photovoltaics demonstrated very limited effects of electron flux ranging from 1 to 100 $e^{-}.Å^{-2}.s^{-1}$.[17]

In the context of LP-TEM of beam-sensitive, low-contrast materials, it is of particular importance to begin with a “safe” dose rate range to minimize significant beam damage while maintaining an acceptable signal-to-noise ratio in the images. From there, we can vary the dose rate and dose to identify the critical dose rate and critical dose (dose budget) and determine the most effective way to use the available dose budget.[1a] In this regard, the manuscript presents the effects of dose rate (from 0.3 to 1.2 $e^{-}.Å^{-2}.s^{-1}$) in Figure 3c and Figure S10,11,19, where electron flux/dose rate effects on water radiolysis are detailed in both water vapor and liquid water experiments. Our findings indicate that within this dose rate range, similar radiolytic damage phenomena occur in the liquid water environment with higher dose rates accelerating the damage process. Intriguingly, the steady-state hydroxyl radical concentration at a given dose rate has been reported to increase likely in a sublinear manner along with the dose rate increase in the absence of diffusion and solutes.[4a] In contrast, in vapor water environments, the effect of dose rates on the dissolution kinetics of polymer stomatocytes are insignificant, since increasing the dose rate by a factor of four has negligible impact on the polymer dissolution process.

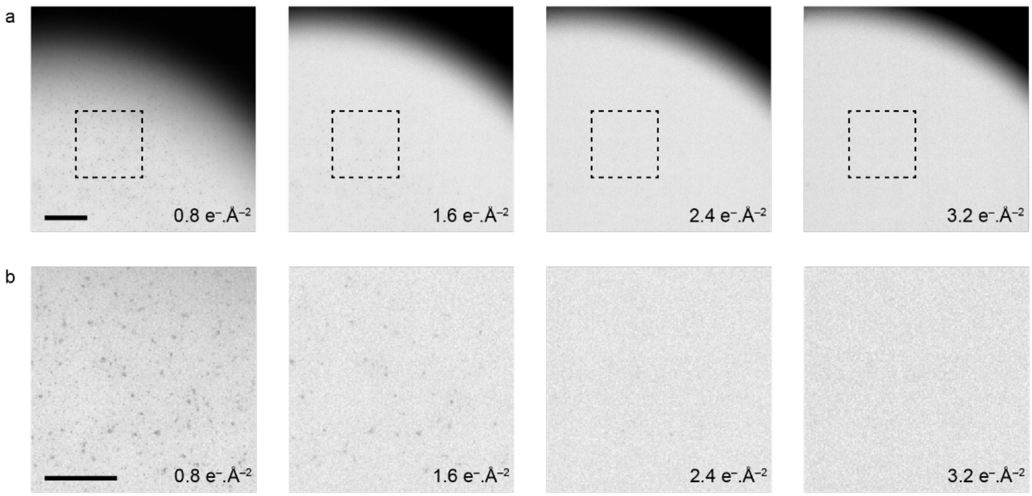

Figure S20. Control experiment showing the importance of the O_2 concentration in radiolytic damage process of polymers. (a) TEM image sequences showing that the nanoparticles previously formed in liquid water rapidly dissolve at the liquid-vapor interface. Electron flux: 0.8 $e^{-}.Å^{-2}.s^{-1}$. (b) TEM image sequences showing the areas highlighted by the boxes in (a). Scale bars: (a) 1 μm. (b) 500 nm.

3.4 Imaging crystalline organic materials in liquid water

3.4.1 Preparation of ZIF-8 nanocrystals

ZIF-8 nanocrystals (NCs) were synthesized using a well-establised hydrothermal method.[18] Typically, zinc nitrate hexahydrate ($Zn(NO_3)_2{\cdot}6H_2O$, 0.4 g) was dissolved in 15 mL of deionized water. Separately, 2-methylimidazole (6.0 g) and cetyltrimethylammonium bromide (CTAB, 0.002 g) were dissolved in 90 mL of DI water. The two solutions were then combined and transferred to a Teflonlined autoclave for reaction at 120 °C for 5 h. After natural cooling to room temperature, the ZIF-8 NCs were collected by centrifugation, washed and dried under vacuum. The resulting ZIF-8 NPs were then characterized by dry-TEM and LP-TEM (Figure S21). The size of the as-synthesized ZIF-8 NCs was measured to be 105 nm ± 15 nm.

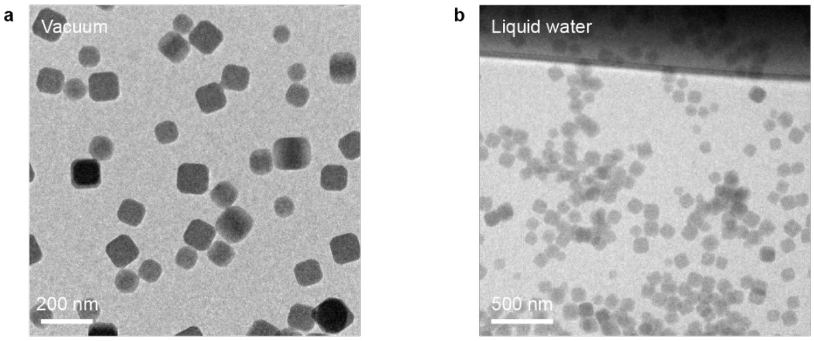

Figure S21. Characterization of as-prepared ZIF-8 NCs using dry-TEM (a) and LP-TEM (b).

3.4.2 Electron diffraction of ZIF-8 nanocrystals in liquid water

In-situ Selected Area Electron Diffraction (SAED) of ZIF-8 NCs (Figure S22-23) in liquid water was performed using the Stream liquid flow cell holder (DENSsolutions B.V.) on a Tecnai T20 (Thermo Fisher Scientific), operated at 200 kV with a tungsten filament. The SAED patterns were recorded using a side-mounted Olympus MegaView G2 camera, with the electron flux maintained at ~0.65 $e^{-}.Å^{-2}.s^{-1}$. We were able to monitor the structural damage of the ZIF-8 framework by electron diffraction in liquid,

as indicated by the intensity changes of {110} Bragg spots (D-spacing: 1.2 nm). Along with the decay in intensity, a displacement of the electron diffraction Bragg spots was also observed, which is likely associated with the sequential loss of crystallinity in ZIF-8.[19] To our surprise, we found that under electron beam irradiation, each ZIF-8 nanocrystal follows a unique radiolytic damage kinetic pathway (Figure S23). The critical dose for four measured ZIF-8 nanoparticles, defined as the relative loss of Bragg spot intensity to 1/e, varied by a few orders of magnitude-differing from previously reported results.[19-20] We speculate that these differences may arise from intrinsic crystal defects introduced during synthesis, as well as variations in local solution chemistry during electron irradiation, with neighboring particles p2 and p3 exhibiting similar damage pathways.

Importantly, we would like to note that performing electron diffraction on liquid samples within a SiN cell filled with aqueous or non-aqueous solutions remains a significant challenge due to the requirement of maintaining an extremely thin encapsulated liquid layer.[8b] Most electron diffraction (ED) measurements on liquid samples reported in the literature are achieved either by utilizing a gas bubble,[21] gradually removing or evaporating the liquid through gas flow,[20, 22] or using a nanochannel fluidic cell design.[5] In this work, we addressed this challenge by employing SiN chips with smaller viewing windows (described in Section 3.3.2) and reducing the overlapping area of each SiN chip's viewing window during liquid cell alignment. This approach allowed us to maintain a fully hydrated SiN cell without creating bubbles.

Gnanasekaran *et al.*[20] recently reported using *in-situ* SAED to monitor radiolytic damage of ZIF-8 NCs in water and dimethylformamide. To acquire sufficient ED signal, they flowed air into the cell to create a thin solvent layer at an electron flux of 3.3 $e^{-}.Å^{-2}.s^{-1}$. In contrast, our method enabled the direct recording of electron diffraction patterns at an electron flux five times lower than that used by Gnanasekaran et al. This allows us to study the structural changes of ZIF-8 within a dose rate range comparable to that used in our amorphous polymer system.

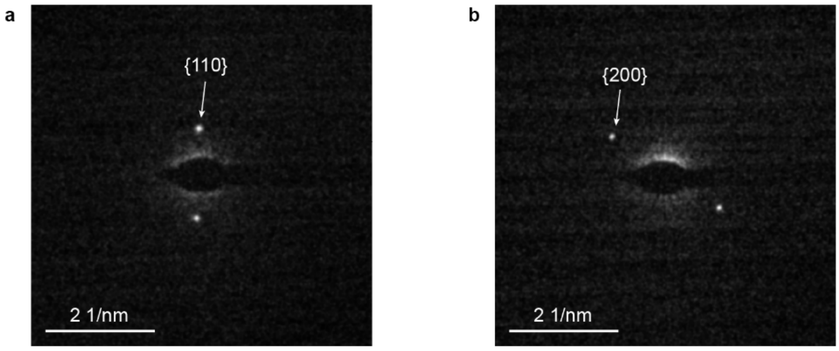

Figure S22. Liquid electron diffraction on ZIF-8 nanoparticles in SiN cells. The presence of {110} Bragg spots in (a) and {200} Bragg spots in (b) within the electron diffraction pattern indicates that information transfer to 1.20 nm and 0.85 nm were achieved in two different SiN cells, each with a much thinner liquid layer. Details of the fabrication of SiN cells with a thin liquid layer suitable for electron diffraction analysis can be found in Section 3.41 of the Supporting Information. Imaging conditions: Electron flux of 0.65 $e^{-}.Å^{-2}.s^{-1}$. Exposure time: 0.2 s/frame.

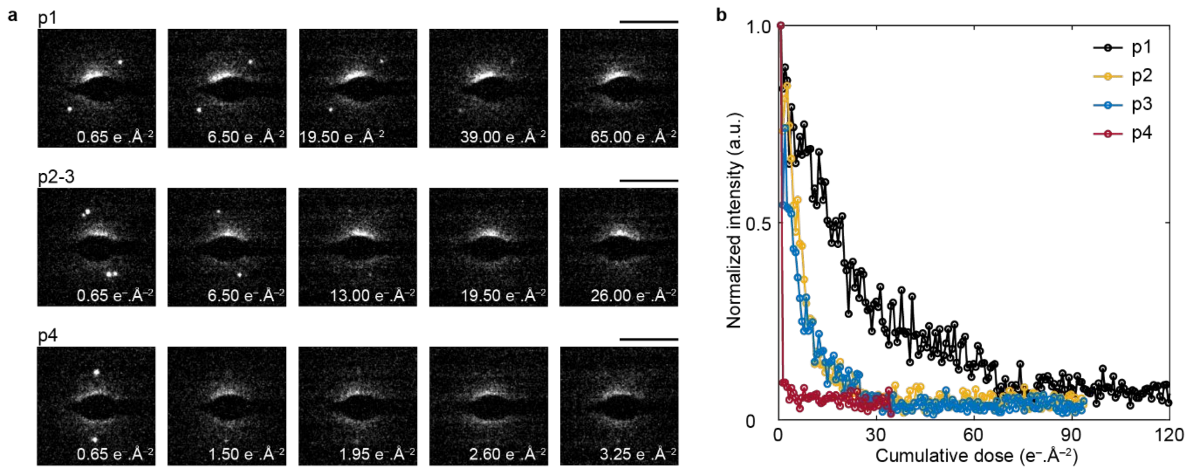

Figure S23. *In-situ* liquid electron diffraction analysis of ZIF-8 nanocrystals. (a) Electron diffraction pattern sequence of four ZIF-8 nanocrystals as a function of cumulative dose. (b) Normalized intensities of the {110} Brag spots as a function of cumulative dose. Electron flux: 0.65 $e^{-}.Å^{-2}.s^{-1}$. Scale bars: 2 1/nm.

3.5 Movie analysis

The movie analysis was predominantly implemented using in-house scripts programmed in MATLAB (MathWorks).[1a, 1b, 2] The key data analysis methodologies are illustrated in Figure S12.

3.5.1 Estimation of liquid layer thickness

We estimated the liquid layer thickness of the prepared liquid cell using a low-dose liquid thickness approximation method that we have previously reported.[1b] The MATLAB source codes for this method are accessible on GitHub.[23]

3.5.2 Alignment of image stacks

In-house MATLAB scripts were utilized to estimate the particle shift between each frame, employing the normalized two-dimensional cross-correlation (N2DCC) method. Details of the particle alignment procedures are available in our previous publication.[1a] After alignment of the image series, an area of interest containing stomatocytes was cropped out for further image analysis.

3.5.3 Determination of relative intensity

To correct for background variation and compare the relative changes in intensity across different images, we normalized the intensity of the entire aligned image stack. The normalization was relative to the average intensity of a background area, ~5 μm away from the region of interest (ROI), which remained free of polymer or nanoparticle deposition throughout the entire movie.[2]

3.5.4 Particle area and intensity tracking

The evolution of projected area and intensity of the stomatocytes under electron irradiation was tracked using in-house MATLAB scripts.[1a] Aligned frames containing single stomatocytes were cropped out, and binarized using Otsu's method. MATLAB's built-in functions *bwareaopen* and *imfill* were employed to remove small objects and fill the remaining holes in the binarized image, respectively. The projected area of each stomatocyte was then quantified by totalling the white pixels in the binary image. To monitor the total intensity evolution within the projected area of a stomatocyte, we firstly segmented individual stomatocytes from the images by multiplying the binary mask with the original image, as illustrated in Figure S12. Subsequently, the cumulative pixel intensity within the stomatocyte area for

each image can be calculated by summing the intensity of all pixels within the projected stomatocyte area.

3.5.5 Estimation of stomatocyte mass change

The mass changes of the stomatocyte are related to the total intensity evolution within their projected area. Since all movies were captured in the bright-field TEM mode, we postulate that the mass of a stomatocyte in frame i is indicative of the total pixel intensity loss ($\sum(1 - I_{x,y}/I_0)$ within its area at frame i, where I_0 represents the average background intensity near the stomatocyte and $I_{x,y}$ denotes the pixel intensity at the coordinate of (x,y) within the stomatocyte, respectively. These intensity loss values were normalized against the value from the first frame and plotted against cumulative dose in Figure S12 and Figure 1 of the main text.

3.5.6 Cross-sectional time series and angular maps

Cross-sectional time series were generated to quantitatively display the morphological evolution of individual stomatocytes under irradiation. This was achieved by displaying a series of 1-pixel-width central line intensity profile from the aligned image stack *versus* time. Additionally, angular maps were used to analyse the local structure of the stomatocyte membrane by plotting intensity maps as a function of angular location around the polymer stomatocyte. Details on the creation of cross-sectional time series and angular maps can be found in our previous publication.[2]

3.6 Kinetic model

We utilized a kinetic model that is widely accepted in the field to compute the concentrations of the primary products of water radiolysis and to track the reaction among these products.[4a, 24] Details of this model are available in previous publications.[4a, 24] To perform the modelling, we adapted MATLAB codes originally developed by Schneider et al.[4a, 25] In our model, we assume that neat water maintains a constant concentration of 55.56 M and is saturated with oxygen at atmospheric conditions, resulting in an O_2 concentration of 0.255 mM. The G-values, which refer to the number of molecules created or destroyed per 100 electronvolts of absorbed energy, are given in Table S2. These values are the same as those for 300 kV electrons,[4a, 24] which were extracted from the graphical data in the study conducted by Hill and Smith.[26] Table S3 lists 83 reactions and their rate constants used in our calculations. Note that most of the reactions and their rate constants are sourced from the work of Schneider et al,[4a] but we also include 10 additional reactions and modify the rate constant of several reactions in light of the recent study by Fritsch et al.[24a]

Table S2. G-Values for 200-300 kV electrons in liquid water[4a, 26]

Primary species	G-Values (molecules/100 eV)
e_h^-	3.47
H^+	4.42
OH^-	0.95
H_2O_2	0.47
H	1.00
OH	3.63
HO_2	0.08
H_2	0.17

Table S3: Rate constants for reactions between radiolysis products of water.[4a, 24a]

	Chemical reaction	Rate constant*
1	$H^+ + OH^- \rightarrow H_2O$	2.60×10^{-5}
2	$H_2O \rightarrow H^+ + OH^-$	1.43×10^{11}
3	$H_2O_2 \rightarrow H^+ + HO_2^-$	1.12×10^{-1}
4	$H^+ + HO_2^- \rightarrow H_2O_2$	5.00×10^{10}
5	$H_2O_2 + OH^- \rightarrow H_2O + HO_2^-$	1.30×10^{10}
6	$HO_2^- + H_2O \rightarrow H_2O_2 + OH^-$	5.82×10^{7}
7	$e_{aq}^- + H_2O \rightarrow H + OH^-$	1.90×10^{1}
8	$H + OH^- \rightarrow e_{aq}^- + H_2O$	2.20×10^{7}
9	$H \rightarrow e_{aq}^- + H^+$	3.90×10^{0}
10	$e_{aq}^- + H^+ \rightarrow H$	2.30×10^{10}
11	$OH + OH^- \rightarrow O^- + H_2O$	1.30×10^{10}
12	$O^- + H_2O \rightarrow OH + OH^-$	1.00×10^{8}
13	$OH \rightarrow O^- + H^+$	1.26×10^{-1}
14	$O^- + H^+ \rightarrow OH$	1.00×10^{11}
15	$HO_2 \rightarrow O_2^- + H^+$	1.35×10^{6}
16	$O_2^- + H^+ \rightarrow HO_2$	5.00×10^{10}
17	$HO_2 + OH^- \rightarrow O_2^- + H_2O$	5.00×10^{10}
18	$O_2^- + H_2O \rightarrow HO_2 + OH^-$	1.86×10^{1}
19	$e_{aq}^- + OH \rightarrow OH^-$	3.00×10^{10}
20	$e_{aq}^- + H_2O_2 \rightarrow OH + OH^-$	1.10×10^{10}
21	$e_{aq}^- + O_2^- + H_2O \rightarrow HO_2^- + OH^-$	1.30×10^{10}
22	$e_{aq}^- + HO_2 \rightarrow HO_2^-$	2.00×10^{10}
23	$e_{aq}^- + O_2 \rightarrow O_2^-$	1.90×10^{10}
24	$2e_{aq}^- + 2H_2O \rightarrow H_2 + 2OH^-$	5.50×10^{9}
25	$e_{aq}^- + H + H_2O \rightarrow H_2 + OH^-$	2.50×10^{10}
26	$e_{aq}^- + HO_2^- \rightarrow O^- + OH^-$	3.50×10^{9}
27	$e_{aq}^- + O^- + H_2O \rightarrow 2OH^-$	2.20×10^{10}
28	$e_{aq}^- + O_3^- + H_2O \rightarrow O_2 + 2OH^-$	1.60×10^{10}
29	$e_{aq}^- + O_3 \rightarrow O_3^-$	3.60×10^{10}
30	$H + H_2O \rightarrow H_2 + OH$	1.10×10^{1}
31	$H + O^- \rightarrow OH^-$	1.00×10^{10}
32	$H + HO_2^- \rightarrow OH + OH^-$	9.00×10^{7}
33	$H + O_3^- \rightarrow OH^- + O_2$	1.00×10^{10}
34	$2H \rightarrow H_2$	7.80×10^{9}
35	$H + OH \rightarrow H_2O$	7.00×10^{9}
36	$H + H_2O_2 \rightarrow OH + H_2O$	9.00×10^{7}
37	$H + O_2 \rightarrow HO_2$	2.10×10^{10}
38	$H + HO_2 \rightarrow H_2O_2$	1.80×10^{10}
39	$H + O_2^- \rightarrow HO_2^-$	1.80×10^{10}
40	$H + O_3 \rightarrow HO_3$	3.80×10^{10}
41	$2OH \rightarrow H_2O_2$	3.60×10^{9}

42	$OH + HO_2 \rightarrow H_2O + O_2$	6.00×10^{9}
43	$OH + O_2^- \rightarrow OH^- + O_2$	8.20×10^{9}
44	$OH + H_2 \rightarrow H + H_2O$	4.30×10^{7}
45	$OH + H_2O_2 \rightarrow HO_2 + H_2O$	2.70×10^{7}
46	$OH + O^- \rightarrow HO_2^-$	2.50×10^{10}
47	$OH + HO_2^- \rightarrow HO_2 + OH^-$	7.50×10^{9}
48	$OH + O_3^- \rightarrow O_3 + OH^-$	2.60×10^{9}
49	$OH + O_3^- \rightarrow 2O_2^- + H^+$	6.00×10^{9}
50	$OH + O_3 \rightarrow HO_2 + O_2$	1.10×10^{8}
51	$HO_2 + O_2^- \rightarrow HO_2^- + O_2$	8.00×10^{7}
52	$2HO_2 \rightarrow H_2O_2 + O_2$	7.00×10^{5}
53	$HO_2 + O^- \rightarrow O_2 + OH^-$	6.00×10^{9}
54	$HO_2 + H_2O_2 \rightarrow OH + O_2 + H_2O$	5.00×10^{-1}
55	$HO_2 + HO_2^- \rightarrow OH + O_2 + OH^-$	5.00×10^{-1}
56	$HO_2 + O_3^- \rightarrow 2O_2 + OH^-$	6.00×10^{9}
57	$HO_2 + O_3 \rightarrow HO_3 + O_2$	5.00×10^{8}
58	$2O_2^- + 2H_2O \rightarrow H_2O_2 + O_2 + 2OH^-$	1.00×10^{2}
59	$O_2^- + O^- + H_2O \rightarrow O_2 + 2OH^-$	6.00×10^{-8}
60	$O_2^- + H_2O_2 \rightarrow OH + O_2 + OH^-$	1.30×10^{-1}
61	$O_2^- + HO_2^- \rightarrow O^- + O_2 + OH^-$	1.30×10^{-1}
62	$O_2^- + O_3^- + H_2O \rightarrow 2O_2 + 2OH^-$	1.00×10^{4}
63	$O_2^- + O_3 \rightarrow O_3^- + O_2$	1.50×10^{9}
64	$2O^- + H_2O \rightarrow HO_2^- + OH^-$	1.00×10^{9}
65	$O^- + O_2 \rightarrow O_3^-$	3.60×10^{9}
66	$O^- + H_2 \rightarrow H + OH^-$	8.00×10^{7}
67	$O^- + H_2O_2 \rightarrow O_2^- + H_2O$	5.00×10^{8}
68	$O^- + HO_2^- \rightarrow O_2^- + OH^-$	4.00×10^{8}
69	$O^- + O_3^- \rightarrow 2O_2^-$	7.00×10^{8}
70	$O^- + O_3 \rightarrow O_2^- + O_2$	5.00×10^{9}
71	$O_3^- \rightarrow O_2 + O^-$	3.30×10^{3}
72	$O_3^- + H^+ \rightarrow O_2 + OH$	9.00×10^{10}
73	$HO_3 \rightarrow O_2 + OH$	1.10×10^{5}
74	$H_2O_2 \rightarrow H_2O + O$	1.00×10^{-3}
75	$2O \rightarrow O_2$	1.00×10^{9}
76	$O_3 \rightarrow O_2 + O$	3.00×10^{-6}
77	$2O_3^- + H_2O \rightarrow OH^- + HO_2^- + 2O_2$	1.00×10^{4}
78	$2HO_3 \rightarrow H_2O_2 + 2O_2$	5.00×10^{9}
79	$O_3 + OH^- \rightarrow HO_2^- + O_2$	1.00×10^{2}
80	$O_2 + O \rightarrow O_3$	4.00×10^{9}
81	$H_2O_2 + O \rightarrow OH + HO_2$	1.60×10^{9}
82	$O + HO_2^- \rightarrow OH + O_2^-$	5.30×10^{9}
83	$O + OH^- \rightarrow HO_2^-$	4.20×10^{8}

* The rate constant has a unit of $mol^{-n+1}L^{3(n-1)}s^{-1}$, where n is the reaction order. Note that these underlined rate constants are sourced from the recent publication by Fritsch et al.[24a]

3.7 Photoirradiation experiments using UV/H_2O_2

3.7.1 Fundamentals of UV/H_2O_2 processes

Under ultraviolet (UV) irradiation, hydrogen peroxide (H_2O_2) can absorb radiation and produce hydroxyl radicals ($OH^•$), a process known as the UV/H_2O_2 process.[27] This method is widely accepted as an easy and controllable way to generate hydroxyl radicals without additional secondary chemical reaction and has been extensively used in water treatment to remove organic pollutants. The photolysis of H_2O_2 is most effectively under UV light in the range of 100 to 280 nm, but H_2O_2 also absorbs some radiation in the range of 315 to 400 nm, albeit with a low molar absorption coefficient above 300 nm.[27a] Typically, each hydrogen molecule produces two $OH^•$ radicals during photolysis, and the their formation kinetics upon hydrogen peroxide have been quantitively described in Ref.[27a, 27c]

The formed $OH^•$ radicals react with other species present in the aqueous solution and generate various radicals. Key reactions and their reaction rate constants are shown in Table S4.[27b, 28] Kinetic modeling of UV/H_2O_2 processes has previously been employed to quantify the effective hydroxyl radicals and to understand the degradation kinetics of pollutant compounds.[27b, 27c, 28] It is worth noting that $OH^•$ can be scavenged by H_2O_2, though the rate constant is relatively low (2.7×10^7 $M^{-1}.s^{-1}$).[27a] Importantly, we note that the rate constants for most reactions used in the kinetic modeling of UV/H_2O_2 processes are either identical or within the same order of magnitude as those used in the kinetic model for water radiolysis in LP-TEM, with the exception of reaction #9 in Table S4.

3.7.2 Determination of hydroxyl radical concentrations using UV-Vis Spectroscopy

The concentration of photochemically generated hydroxyl radicals in the UV/H_2O_2 process can be measured by various methods reported previously.[29] In this study, we used salicylic acid (SA), a well-known $OH^•$ probe molecule, to quantify the steady-state hydroxyl radical concentration, $C_{ss,OH^•}$. SA reacts with $OH^•$ radicals to form a mixture of 2,3-dihydroxybenzoic acid (23DA) and 2,5-dihydroxybenzoic acid (25DA) in solution (Scheme S2). Given that the reaction rate constant between SA and $OH^•$ is known ($k_{SA,OH^•}$, 5×10^9 $M^{-1}s^{-1}$), $C_{ss,OH^•}$ can be determined by quantifying either the decomposition rate of parent SA or the formation rate of 23DA and 25 DA. The concentration of SA, 23 DA, and 25 DA at different UV exposure times can be measured by UV-Vis spectroscopy (see details in Ref.[29a]), allowing the determination of SA's loss rate. Lankone et al[29b] reported that the decomposition of SA follows the pseudo-first-order kinetics with a rate constate of k_{SA}, and $C_{ss,OH^•}$ can be estimated as:

$$C_{ss,OH^•} = \frac{k_{SA}}{k_{SA,OH^•}} \tag{3}$$

To experimentally determine hydroxyl radical concentrations under the UV conditions used in our experiments, we prepared a sample solution containing SA (0.07 mM) and hydrogen peroxide (0.02 wt%, ~6 mM) in the absence of polymers. Since the UV-Vis absorbance peaks of hydrogen peroxide can overlap with those of SA and its reaction products, here we used a much lower hydrogen peroxide concentration. A 2.5 mL sample solution was then transferred to a 20 mL vial and placed inside a sealed photor-eactor chamber equipped with a S1000 OmniCure mercury spot UV curing lamp (S1000, OmniCure, 320-500 nm) connected to EXFO fiber optic light guides. After purging the system with nitrogen for 30 min, the sample solution was exposed to UV light and , and aliquots were collected at

different exposure times and analyzed using a Cary 3500 UV-Vis spectrophotometer (Agilent). The steady-state concentration of generated hydroxyl radicals, $C_{ss,OH^{\bullet}}$, was estimated to be on the order of 10^{-12} M, based on the methods described above (Figure S24). It is important to note that the wavelength range used in this study is not optimal, and the corresponding molar absorption coefficient in this range is likely quite low.[27a] Adjusting the concentration of H_2O_2 and the UV wavelength may allow us to achieve hydroxyl radical concentrations similar to those predicted by our water radiolysis modeling. But we have noted that at high concentrations of H_2O_2, the UV-Vis absorbance peaks of H_2O_2 can overlap with those of SA and its reaction products, complicating the measurements. Optimizing the UV wavelength may help but remain a future research objective beyond the scope of this study.

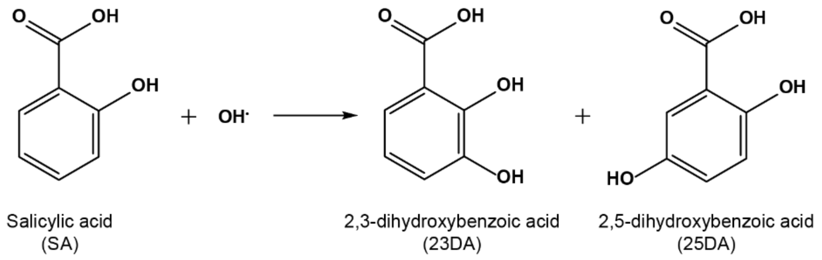

Scheme S2. Reaction between salicylic acid (SA) and hydroxyl radicals, leading to the formation of 2,3-dihydroxybenzoic acid (23DA) and 2,5-dihydroxybenzoic acid (25DA).

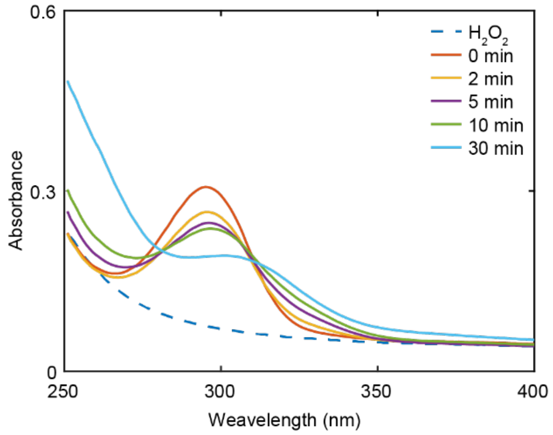

Figure S24. UV-Vis spectra as a function of reaction time. Absorbances were measured at 290, 310 and 330 nm, which correspond to the maximum absorbance wavelengths[29a] of SA, 23DA and 25 DA, respectively, at different reaction times. The initial concentrations of salicylic acid (SA) and H_2O_2 are 0.07 mM and 0.02 wt%, respectively.

Table S4: Key chemical reactions involved in the UV/H_2O_2 process[a][27b, 27c, 28]

	Reactions	Rate constants ($M^{-1}s^{-1}$)[b]	Reference
1	$H_2O_2 + hv \rightarrow 2OH$	ε = 18.6 $M^{-1}cm^{-1}$, Φ = 0.5 mol/E	[30]
2	$HO_2^- + H_2O + hv \rightarrow 2OH + OH^-$	ε = 228 $M^{-1}cm^{-1}$, Φ = 0.5 mol/E	[31]
3	$OH + P \rightarrow Products$	To be determined	
4	$OH + HO_2^- \rightarrow HO_2 + OH^-$	7.5×10^{9}	[32]
5	$OH + H_2O_2 \rightarrow HO_2 + H_2O$	2.7×10^{7}	[33]
6	$OH + HO_2 \rightarrow H_2O + O_2$	$\underline{6.6\times10^{9}}$	[34]
7	$OH + O_2^- \rightarrow O_2 + OH^-$	$\underline{7.0\times10^{9}}$	[33]
8	$2OH \rightarrow H_2O_2$	$\underline{5.5\times10^{9}}$	[33]
9	$HO_2 + O_2^- \rightarrow HO_2^- + O_2$	$\underline{9.7\times10^{7}}$	[35]
10	$HO_2 + H_2O_2 \rightarrow OH + H_2O + O_2$	$\underline{2.5\text{-}5.0\times10^{0}}$	[33]
11	$O_2^- + H_2O_2 \rightarrow OH + OH^- + O_2$	1.3×10^{-1}	[36]
12	$2HO_2 \rightarrow H_2O_2 + O_2$	$\underline{8.3\times10^{5}}$	[35]
13	$H_2O_2 \Leftrightarrow HO_2^- + H^+ (pKa = 11.6)$	k_{for} = 2.51×10^{-2}, k_{back} = 1.0×10^{10}	[37]
14	$HO_2 \Leftrightarrow O_2^- + H^+ (pKa = 4.8)$	k_{for} = 1.58×10^{5}, k_{back} = 1.0×10^{10}	[37]
15	$H_2O \Leftrightarrow OH^- + H^+ (pKa = 16.0)$	k_{for} = 1.14×10^{-6}, k_{back} = 1.0×10^{10}	[27b]

[a] Chemical reactions involving nitrate or carbonate ions can be found in the Ref.[27b, 27c, 28]. Here, ε denotes the molar absorption of the compound coefficient, and Φ is the quantum yield. P refers to the polymer under study and $Products$ include the reaction products, such as polymer macroradicals formed by hydrogen abstraction (H-abstraction) by hydroxyl radicals, and those resulting from cross-linking and scission, followed by H-abstraction.[38] Reactions between polymer macroradicals and O_2 are not included here. k_{for} and k_{back} denote the rates of forward and back reactions, respectively.[27b]

[b] Some rate constants are underlined to indicate that they are different from those for the same reactions listed in Table S3.

3.7.3 Mimicking radiolytic damage of stomatocytes using UV/H_2O_2

First, 0.5 g of stomatocyte solution (2.85 mg/ml) and 2 g H_2O_2 (12.5-20 wt%) were added to a 20 mL vial. The glass vial was then placed in a sealed photo-reactor chamber equipped with a S1000 OmniCure mercury spot UV curing lamp (S1000, OmniCure, 320-500 nm) with EXFO fiber light guides.[39] The mixed dispersion was stirred at 1600 rpm under the nitrogen gas flow and samples were collected for Cryo-TEM (Figure S25), dry-TEM (Figure S27), DLS, MALDI-MS (Figure S28) and GPC measurements after different exposure times. Samples were lyophilized for 12 h after UV exposure prior to MALDI-MS and GPC measurements.

Furthermore, we have also quantitatively analyzed the nanoparticles formed in both LP-TEM and UV/H_2O_2 experiments. Under UV irradiation, the nanoparticles had an average size of 5.04 ± 0.46 nm. In LP-TEM, most initially formed nanoparticles on the stomatocyte had sizes typically ranging from 5.34 nm to 8.01 nm, corresponding to 2 to 3 pixels in the LP-TEM data. These small nanoparticles in LP-TEM were observed to grow and coalesce to form larger patches, even at a cumulative dose of 1.5 $e^-.Å^{-2}$, as shown in Figure S26a. In contrast, the largest nanoparticles observed under UV irradiation, characterized via cryo-TEM, were approximately 6 nm in size. We speculate that this 6 nm size represents a critical threshold, where PEG chain scission reduces stomatocyte solubility, leading to significant stomatocyte aggregation. These aggregates become too large to be vitrified and characterized by cryo-TEM, although similar final products can still be observed after extensive UV irradiation. The

comparable sizes of nanoparticles formed in both LP-TEM and UV/H_2O_2 experiments suggest that we have successfully replicated similar radiolytic damage to polymers outside the electron microscope. We recognize the need for further optimization to improve our comparative analysis through kinetically modelling of polymer degradation in both LP-TEM and UV/H_2O_2 process.

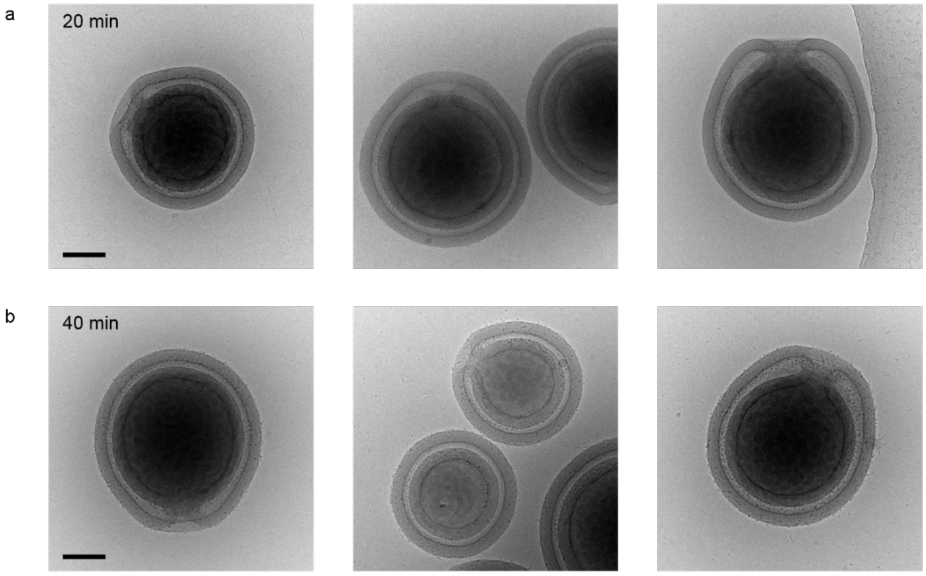

Figure S25. Cryo-TEM confirmed the formation of nanoparticles on the stomatocyte membrane surface at different UV irradiation times in the presence of H_2O_2. (a) 20 min. (b) 40 min. Scale bars: 100 nm.

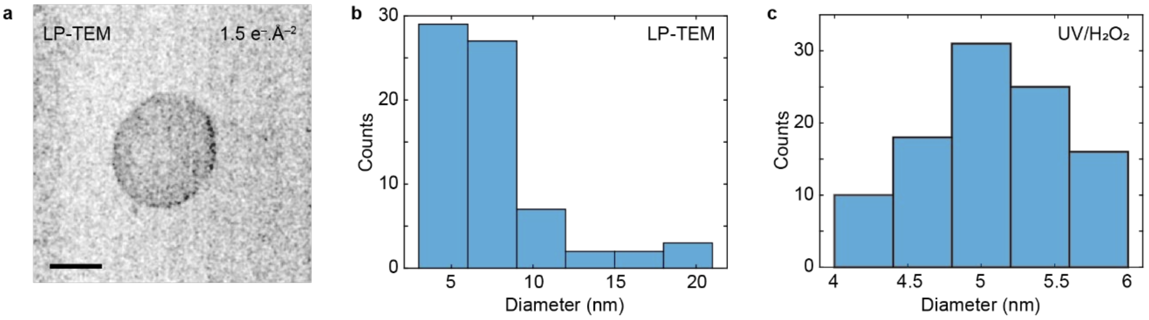

Figure S26. Size distribution of nanoparticles formed in LP-TEM and UV/H_2O_2 experiments. (a) LP-TEM image of a polymer stomatocyte (5-frame average) at a cumulative dose of 1.5 $e^{-}.Å^{-2}$. Scale bar: 200 nm. (b-c) Histograms showing the size distribution of nanoparticles formed in LP-TEM (b) and in UV/H_2O_2 experiments (c). A total of 70 nanoparticles were measured from the LP-TEM image, and 100 were measured from the cryo-TEM images shown in Figure S25. The size measurement was carried out using the line profile tool in the Digital Micrograph software.

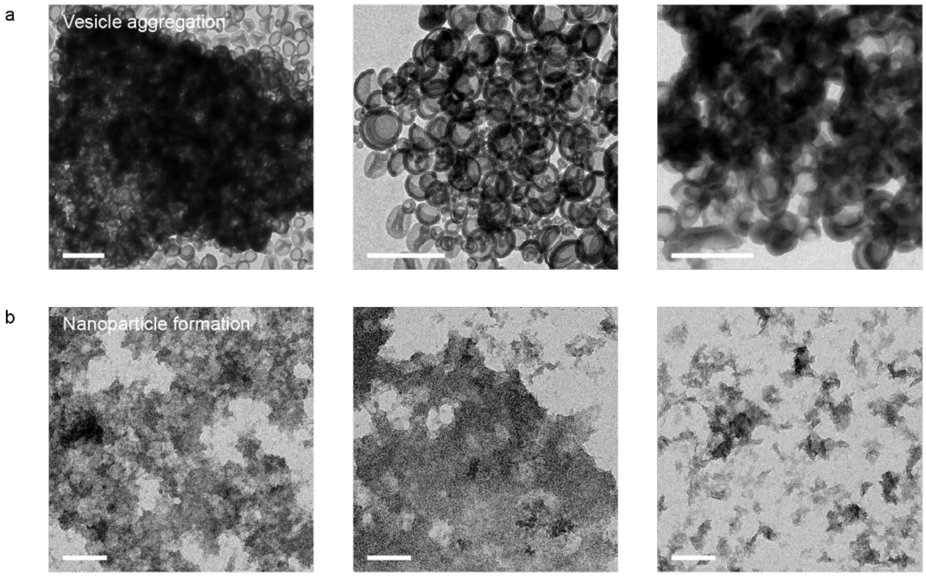

Figure S27. Dry-TEM characterization of lyophilized samples of the foam layer and liquid layer after 4 h UV irradiation. (a) TEM images showing the aggregation of stomatocytes. (b) TEM images showing the formation of nanoparticles after UV irradiation. Scale bars: (a) 1 μm, (b) 100 nm.

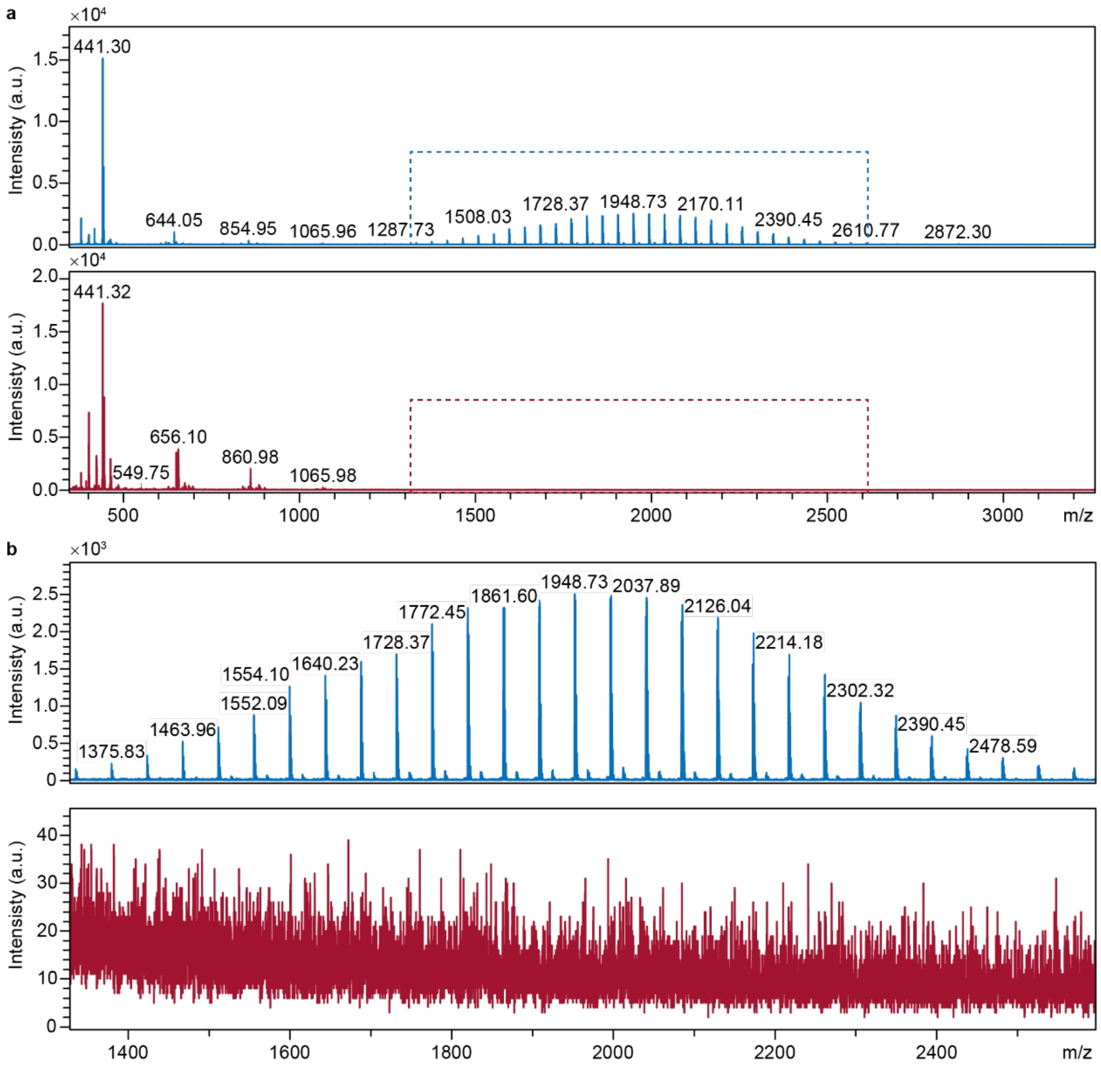

Figure S28. Control MALDI-MS experiment showing that pure PEG_{2K} polymers can be completely degraded to low molecular weight compounds under the same UV/H_2O_2 conditions. (a) Mass spectra of PEG before (blue) and after (red) UV irradiation in the presence of H_2O_2. (b) Zoomed mass spectra of PEG from the regions highlighted in (a).

3.8 Graphene-coated SiN cell and graphene liquid cell

3.8.1 Graphene-coated SiN cell

Multilayer graphene layers were transferred to the top chip of the STREAM liquid cell (DENSsolutions B.V.) using a modified graphene transfer method based on existing techniques.[40] This optimized method ensures full wafer coverage while minimizing contamination by metals or polymers. Electron diffraction and Raman spectroscopy confirmed the transfer of large-area, high-quality and clean multilayer graphene layers onto the top chip of the liquid cell (Figure S29-30). The sample loading method is the same as described in section 3.3.2.

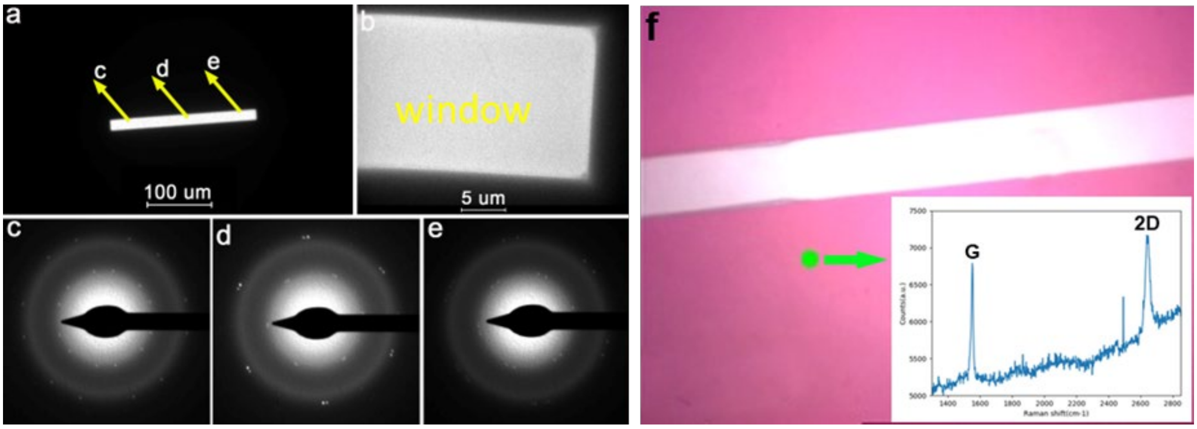

Figure S29. Characterizations of the graphene coated top chip. (a) Low- and (b) high-magnification TEM bright-field images, (c-e) Selected area electron diffraction (SAED) patterns on the corresponding positions on the amorphous SiN membrane depicted in (a). (f) Optical image and the Raman spectrum (inset) measured under 6.2 mW excitation of 632.8 nm He-Ne laser line focused on the green spot away from the membrane.

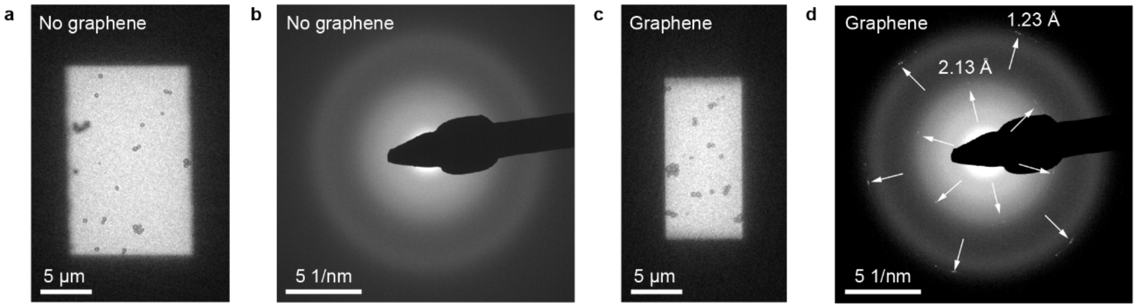

Figure S30. Verifying the presence of the coated graphene in the assembled liquid cells using SAED. (a, c) Overview of the liquid cell and corresponding SAED pattern without (a, b) and with (c, d) the coated graphene after loading ~1.5 nL aliquot of PEG-*b*-PS stomatocyte solution using a picolitre dispensing device. The white arrows in (d) indicate the diffraction spots of the multilayer graphene.

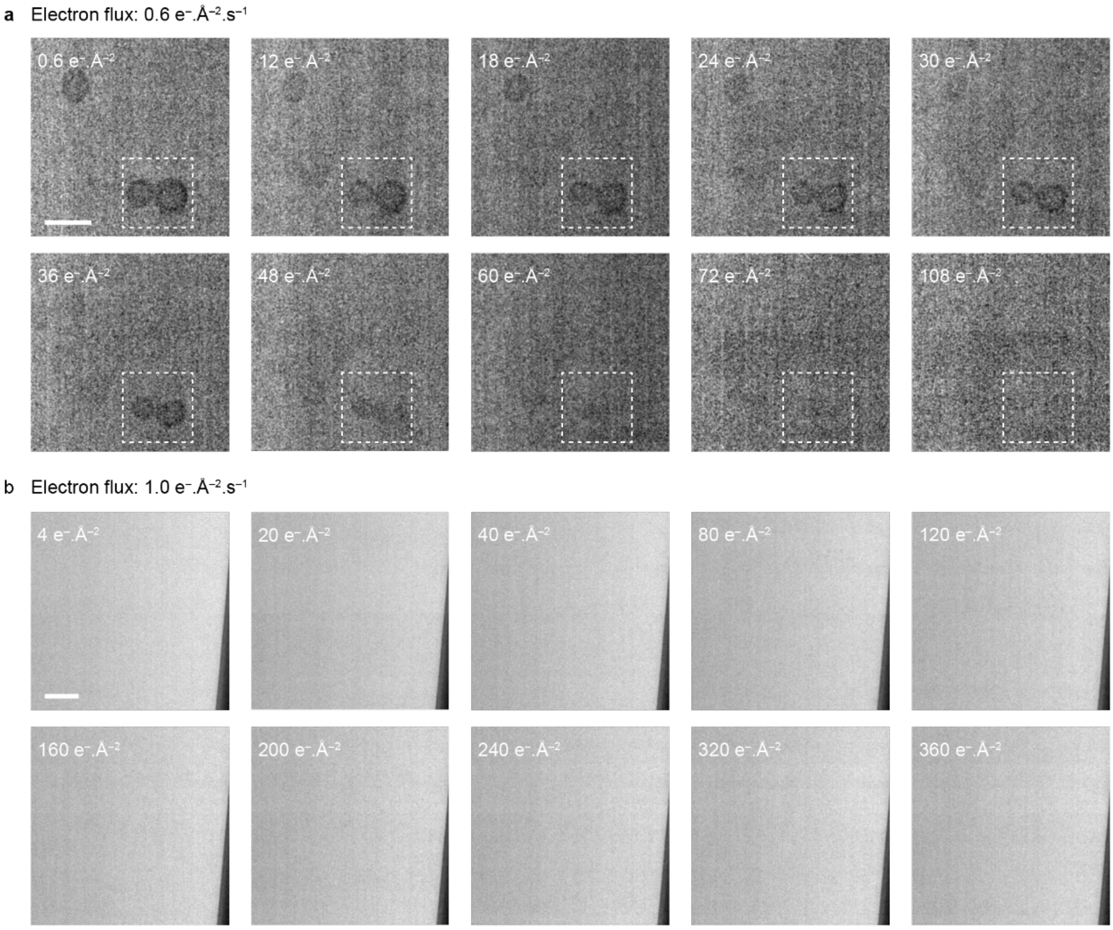

Figure S31. LP-TEM imaging of stomatocytes in water with multilayer graphene on the top chip to protect the polymer sample. (a) LP-TEM image sequence showing the dissolution of polymer stomatocytes at an electron flux of 0.6 e$^-$.Å^{-2}.s^{-1}. The white dashed boxes highlight two stomatocytes shown in Figure 4b in the main text. (b) LP-TEM image sequence showing the absence of nanoparticle formation on the SiN membrane even at an electron flux of 1.0 e$^-$.Å^{-2}.s^{-1}. Scale bars: 500 nm.

3.8.2 Graphene liquid cell (GLC)

Graphene liquid cells (GLCs) were fabricated according to previous reports.[41] A monolayer graphene film on copper (Sigma) was first cut into 2×2 mm squares and the copper was etched in 0.1 M ammonium persulfate (APS, ≥98%, Sigma) aqueous solution. When the copper substrate was removed, the APS solution was gradually replaced with MilliQ water (10 times). 1 μL of the stomatocyte solution was then pipetted onto a gold TEM grid with a continuous carbon film (rendered hydrophilic by 40 s oxygen plasma), after which the clean floating graphene was immediately transferred to the TEM grid using a homemade metal loop and left to dry at room temperature for 12 h.

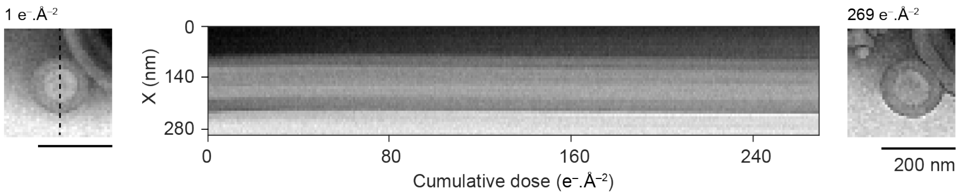

Figure S32. Cross-sectional dose series showing the bilayer membrane remained unchanged during electron irradiation.

3.8.3 Roadmap for studying polymers in liquids using LP-TEM

Given the significant challenges in imaging beam sensitive polymers in (non)aqueous liquids using LP-TEM, more systematic and rational experimental approaches are needed that yield interpretable data and answer specific research questions in polymer science.[8b, 13a, 38] In first instance we recommend following the decision tree (Figure 7 in Ref.[8b]) to assess whether LP-TEM is suitable for the research question at hand and to guide the selection of optimal experimental conditions. Below, we expand on this by providing a roadmap of key steps for successfully studying polymers/organics in LP-TEM, along with guidance on selecting the most suitable experimental setup for a specific polymer system.

(1) Conceptualizing a research question for LP-TEM

Key question: *Is the research question of interest solvable by LP-TEM?*

In this step, the researchers should first evaluate whether the required spatial resolution and temporal resolution can be achieved by LP-TEM based on the sample information acquired from other morphology characterization techniques. The feature of interest should fall within LP-TEM's spatial resolution range for polymer systems in the SiN cell (1-10 nm), and the temporal resolution (0.01-100 s) should capture the feature's evolution.[13a] To ensure a reasonable spatial resolution, the sample thickness should be less than 1 μm. Researchers should also ensure the compatibility between the sample and LP-TEM hardware, including microchips (Si, SiN, adhesion layer and passivation layer), O-rings and the tubing (PEEK, silica). For experiments involving organic solvents, solvent-resistant O-rings should be used instead of standard Viton O-rings to prevent sealing issues and contamination.

(2) Selecting a liquid cell[8b]

Key question: *Which type of liquid cell best mimics bulk solution conditions?*

Each liquid cell design has its own advantages and disadvantages, and the choice depends on the available liquid holder a researcher has access to. If possible, it is always recommended to compare results from different liquid cells. The guidelines for selecting liquid cells (static cells/SiN flow cells/graphene liquid cells) can be found in Table 1 in Ref.[8b]. The material, thickness and dimensions of the viewing window should be chosen to maximize the signal to background ratio. For slow-reaction kinetics, such as dialysis, a bypass flow cell with a thin spacer is recommended for liquid mixing/exchange. For reactions requiring fast liquid exchange, direct-flow or diffusion cells are more suitable, though rapid changes in the chemical environment may occur between the window membrane.

(3) Determination of dose budget

Key questions: *Which is the critical dose threshold of the polymer sample in liquid at low dose rate conditions with/without the radical scavengers? Does the selected scavenger react with the polymer sample?*

For amorphous polymers, the critical dose can be estimated by monitoring beam-induced changes in size, morphology or intensity. The critical dose is defined as the dose at which significant changes have occurred, *e.g.* changes in size, morphology or intensity.[1a] For crystalline polymers, electron diffraction can be used by measuring the intensity decay of Bragg spots until 1/e, though this requires an extremely thin liquid layer. To increase the dose budget, radical scavengers or controlling physical parameters are often used, as outlined in Table S5. It's important to assess the compatibility of the sample with the scavenger and if the sample has scavenger like components in Table S5, it is expected that they will be degraded too. We refer readers to the review article by Woehl and Abellan[15] for further information on

this topic. In general, heavy water, graphene quantum dots or graphene sheets are recommended. For alcohol-based scavengers, concentration should be optimized based on the samples' compatibility.[24b]

(4) Rational distribution of the dose budget

Key question: *What are the optimal frame number and dose per frame for this polymer system?*

Once the critical dose, or dose budget, is determined, it can be distributed across frames to optimize the signal-to-noise ratio of each frame. Based on the Rose criterion, the SNR should exceed a value of 3-5.[13a] Depending on the reaction kinetics of a specific polymer system, the temporal distribution of the dose should be adjusted to favor the times where the polymer sample is changed the most. Note that the beam sensitive of the polymer may also change during reactions.

(5) Validating LP-TEM observations

Key question: *What are the roles of beam and confinement effects on LP-TEM data?*

Validation of acquired LP-TEM data is critical and it is recommended to perform a range of control experiments with various dose rates, spacer thicknesses, comparing the results with data obtained by other complimentary techniques, such as cryo-TEM or scattering. Post-mortem analysis on the liquid cells is also recommended. More details can be found in Section 3.3.3.

Table S5. Overview of beam damage control strategies using radical scavengers and physical parameters[15]

	Radicals		Properties	
	$OH^\bullet$	e_h^-	**Native solution chemistry alteration**	**Preparation time[a]**
**Radical scavengers[b]**				
Alcohol ($-CH_2OH$)	✓		✓	low
Saturated carbon ($-CH_2-$)	✓		✓	low
Halide Ions (Br^-, Cl^-, I^-)	✓		✓	low
Amine ($-CH_2$)	✓		✓	low
Ether (−O−)	✓		✓	low
Alkene	✓		✓	low
Aromatic	✓		✓	low
O_2		✓	△	low
H^+		✓	✓	low
Nitro ($-NO_2$)		✓	✓	low
Bromo (−Br)		✓	✓	low
Iodo (−I)		✓	✓	low
Graphene sheets	✓		×	high
Graphene quantum dots	✓		×	low
D_2O[c]	✓	✓	△	low
**Control of physical parameters[d]**				
Low dose imaging	✓	✓	✓	low
High accelerating voltage	✓	✓	✓	low
Flow control	△	△	△	high
Cell thickness control	✓	✓	✓	high

[a] Preparation time includes introducing the radical scavengers, assembling the liquid cell and controlling physical parameters; [b] Adding radical scavengers in LP-TEM generally alters the native chemical environment and can vary with concentration. Note that some polymers may contain the organic functional groups listed in this Table;[15] [c] Deuterated water (D_2O) is not usually classified as a radical scavenger, but its slower radiolysis compared to H_2O leads to reduced production of reactive radicals and bubbles.[42] [d] Adjusting physical parameters like dose rates, accelerating voltage, and liquid thickness can influence water radiolysis.[8b] Symbols used: ✓= Yes, ×= No, △= Case dependent.

4. Captions of Supplementary Movies

Movie S1. Movie showing electron beam damage to polymer stomatocytes under three different environments. Electron flux: 0.6 $e^-.Å^{-2}.s^{-1}$.Acquisition frame rate: 1 fps. Playback frame rate: 10 fps.

Movie S2. LP-TEM movie showing several stomatocytes exposed to electron irradiation in a thick water layer. Electron flux: 0.6 $e^-.Å^{-2}.s^{-1}$. Acquisition conditions: 1 seconds/frame. Playback frame rate: 50 fps.

Movie S3. LP-TEM movie showing how early-stage damage occurrs on the polymer stomatocytes. Electron flux: 0.3 $e^-.Å^{-2}.s^{-1}$. Acquisition conditions: 1 seconds/frame. Playback frame rate: 40 fps.

Movie S4. LP-TEM movie showing how preformed dendritic nanoparticles attach to a damaged stomatocyte and how a trapped air bubble escapes from the cavity of the stomatocyte upon electron irradiation. Acquisition conditions: 1 second/frame. Playback frame rate: 40 fps. Electron flux: 0.6 $e^{-}.Å^{-2}.s^{-1}$.

Movie S5. LP-TEM movie showing the formation and aggregation process of dendritic nanoparticles in the solution. Electron flux: 0.6 $e^{-}.Å^{-2}.s^{-1}$. Acquisition conditions: 1 seconds/frame. Playback frame rate: 40 fps.

Movie S6. LP-TEM data of imaging polymer stomatocytes in a liquid cell with 5 v% IPA. Electron flux: 0.6 $e^{-}.Å^{-2}.s^{-1}$. Scale bar: 1 μm. Acquisition conditions: 2 seconds/frame. Playback frame rate: 15 fps.

Movie S7. LP-TEM data of imaging polymer stomatocytes in a liquid cell coated with multilayer graphene on the top chip. Electron flux: 0.6 $e^{-}.Å^{-2}.s^{-1}$. Acquisition conditions: 1 second/frame. Playback frame rate: 40 fps.

Movie S8. LP-TEM data of imaging polymer stomatocytes encapsulated inside a graphene cell. Electron flux: 0.85 $e^{-}.Å^{-2}.s^{-1}$. Acquisition conditions: 1 second/frame. Playback frame rate: 40 fps.

5. References

[1] a)H. Wu, T. Li, S. P. Maddala, Z. J. Khalil, R. R. M. Joosten, B. Mezari, E. J. M. Hensen, G. de With, H. Friedrich, J. A. van Bokhoven, J. P. Patterson, *ACS Nano* **2021**, 15, 10296; b)H. Wu, H. Su, R. R. Joosten, A. D. Keizer, L. S. van Hazendonk, M. J. Wirix, J. P. Patterson, J. Laven, G. de With, H. Friedrich, *Small Methods* **2021**, 2001287; c)J. T. van Omme, H. Wu, H. Y. Sun, A. F. Beker, M. Lemang, R. G. Spruit, S. P. Maddala, A. Rakowski, H. Friedrich, J. P. Patterson, H. H. P. Garza, *J. Mater. Chem. C.* **2020**, 8, 10781.

[2] A. Ianiro, H. Wu, M. M. J. van Rijt, M. P. Vena, A. D. A. Keizer, A. C. C. Esteves, R. Tuinier, H. Friedrich, N. Sommerdijk, J. P. Patterson, *Nat. Chem.* **2019**, 11, 320.

[3] S. A. Meeuwissen, K. T. Kim, Y. C. Chen, D. J. Pochan, J. C. M. van Hest, *Angew. Chem. Int. Ed.* **2011**, 50, 7070.

[4] a)N. M. Schneider, M. M. Norton, B. J. Mendel, J. M. Grogan, F. M. Ross, H. H. Bau, *J. Phys. Chem. C* **2014**, 118, 22373; b)B. Fritsch, A. Hutzler, M. J. Wu, S. Khadivianazar, L. Vogl, M. P. M. Jank, M. März, E. Spiecker, *Nanoscale Advances* **2021**, 3, 2466.

[5] M. N. Yesibolati, S. Lagana, S. Kadkhodazadeh, E. K. Mikkelsen, H. Y. Sun, T. Kasama, O. Hansen, N. J. Zaluzecb, K. Molhave, *Nanoscale* **2020**, 12, 20649.

[6] Y. H. Wang, D. Rastogi, K. Malek, J. Y. Sun, A. Asa-Awuku, T. J. Woehl, *J. Phys. Chem. A* **2023**, 127, 2545.

[7] W. Wang, T. Xu, J. G. Chen, J. Shangguan, H. Dong, H. S. Ma, Q. B. Zhang, J. W. Yang, T. T. Bai, Z. R. Guo, H. P. Fang, H. M. Zheng, L. T. Sun, *Nat. Mater.* **2022**, 21, 859.

[8] a)L. R. Parent, E. Bakalis, M. Proetto, Y. W. Li, C. Park, F. Zerbetto, N. C. Gianneschi, *Acc. Chem. Res.* **2018**, 51, 3; b)H. Wu, H. Friedrich, J. P. Patterson, N. A. Sommerdijk, N. de Jonge, *Advanced Materials* **2020**, 32, 2001582, 2001582; c)L. R. Parent, E. Bakalis, A. Ramirez-Hernandez, J. K. Kammeyer, C. Park, J. J. de Pablo, F. Zerbetto, J. P. Patterson, N. C. Gianneschi, *J. Am. Chem. Soc.* **2017**, 139, 17140; d)M. N. Yesibolati, K. I. Mortensen, H. Sun, A. Brostrøm, S. Tidemand-Lichtenberg, K. Mølhave, *Nano Lett.* **2020**, 20, 7108.

[9] A. Verch, M. Pfaff, N. de Jonge, *Langmuir* **2015**, 31, 6956.

[10] S. Merkens, C. Tollan, G. De Salvo, K. Bejtka, M. Fontana, A. Chiodoni, J. Kruse, M. A. Iriarte-Alonso, M. Grzelczak, A. Seifert, *Nat. Commun.* **2024**, 15, 2522.

[11] E. A. Ring, N. de Jonge, *Micron* **2012**, 43, 1078.

[12] M. A. Touve, A. S. Carlini, N. C. Gianneschi, *Nat. Commun.* **2019**, 10, 1.

[13] a)N. de Jonge, L. Houben, R. E. Dunin-Borkowski, F. M. Ross, *Nat. Rev. Mater.* **2019**, 4, 61; b)N. de Jonge, *Ultramicroscopy* **2018**, 187, 113.

[14] L. Reimer, *Transmission electron microscopy: physics of image formation and microanalysis*, Vol. 36, Springer, **2013**.
[15] T. Woehl, P. Abellan, *Journal of microscopy* **2017**, 265, 135.
[16] H. Wu, Doctor of Philosophy, Technische Universiteit Eindhoven, Eindhoven 2020.
[17] Z. J. Leijten, A. D. Keizer, G. de With, H. Friedrich, *The Journal of Physical Chemistry C* **2017**.
[18] Y. H. Zhu, J. Ciston, B. Zheng, X. H. Miao, C. Czarnik, Y. C. Pan, R. Sougrat, Z. P. Lai, C. E. Hsiung, K. X. Yao, I. Pinnau, M. Pan, Y. Han, *Nat. Mater.* **2017**, 16, 532.
[19] P. Banerjee, K. L. Kollmannsberger, R. A. Fischer, **2024**.
[20] K. Gnanasekaran, N. D. Rosenmann, R. dos Reis, N. C. Gianneschi, *Nano Lett.* **2024**, 24, 10161.
[21] Y. Yang, S. Louisia, S. Yu, J. B. Jin, I. Roh, C. B. Chen, M. V. F. Guzman, J. Feijóo, P. C. Chen, H. S. Wang, C. J. Pollock, X. Huang, Y. T. Shao, C. Wang, D. A. Muller, H. D. Abruña, P. D. Yang, *Nature* **2023**, 614, 262.
[22] Y. Pivak, J. Park, S. Basak, R. A. Eichel, A. Beker, A. Rozene, H. H. P. Garza, H. Y. Sun, *Microscopy* **2023**, 72, 520.
[23] https://github.com/hwuNL/Liquid-thickness-mapping-in-LPTEM.
[24] a)B. Fritsch, A. Körner, T. Couasnon, R. Blukis, M. Taherkhani, L. G. Benning, M. P. Jank, E. Spiecker, A. Hutzler, *J. Phys. Chem. Lett.* **2023**, 14, 4644; b)J. Korpanty, L. R. Parent, N. C. Gianneschi, *Nano Lett.* **2021**, 21, 1141.
[25] https://github.com/NMSchneider/Radiolysis.
[26] M. Hill, F. Smith, *Radiat. Phys. Chem.* **1994**, 43, 265.
[27] a)S. Gligorovski, R. Strekowski, S. Barbati, D. Vione, *Chem. Rev.* **2015**, 115, 13051; b)P. Mazellier, É. Leroy, J. De Laat, B. Legube, *New J. Chem.* **2002**, 26, 1784; c)A. Rubio-Clemente, E. Chica, G. A. Peñuela, *Physico-Chemical Wastewater Treatment Resource Recovery* **2017**, 19.
[28] B. A. Wols, D. J. H. Harmsen, E. F. Beerendonk, C. H. M. Hofman-Caris, *Chem. Eng. J.* **2014**, 255, 334.
[29] a)E. Peralta, G. Roa, J. A. Hernandez-Servin, R. Romero, P. Balderas, R. Natividad, *Electrochim. Acta* **2014**, 129, 137; b)R. S. Lankone, A. R. Deline, M. Barclay, D. H. Fairbrother, *Talanta* **2020**, 218, 121148.
[30] D. H. Volman, J. C. Chen, *J. Am. Chem. Soc.* **1959**, 81, 4141.
[31] J. Baxendale, J. Wilson, *Trans. Faraday Society* **1957**, 53, 344.
[32] H. Christensen, K. Sehested, H. Corfitzen, *The Journal of Physical Chemistry* **1982**, 86, 1588.
[33] G. V. Buxton, C. L. Greenstock, W. P. Helman, A. B. Ross, *J. Phys. Chem. Ref. Data* **1988**, 17, 513.
[34] K. Sehested, O. L. Rasmussen, H. Fricke, *The Journal of Physical Chemistry* **1968**, 72, 626.
[35] B. H. Bielski, D. E. Cabelli, R. L. Arudi, A. B. Ross, *Journal of physical chemical reference data* **1985**, 14, 1041.
[36] J. Weinstein, B. H. J. J. o. t. A. C. S. Bielski, *J. Am. Chem. Soc.* **1979**, 101, 58.
[37] R. H. Perry, D. W. Green, J. O. Maloney, *Perry's chemical engineers' handbook*, Vol. 19984, McGraw-Hill New York, **1984**.
[38] L. R. Parent, K. Gnanasekaran, J. Korpanty, N. C. Gianneschi, *ACS Macro Lett.* **2021**, 10, 14.
[39] S. Y. Li, L. G. J. van der Ven, A. B. Spoelstra, R. Tuinier, A. C. C. Esteves, *J. Colloid Interface Sci.* **2023**, 646, 185.
[40] S. Ullah, X. Q. Yang, H. Q. Ta, M. Hasan, A. Bachmatiuk, K. Tokarska, B. Trzebicka, L. Fu, M. H. Rummeli, *Nano Research* **2021**, 14, 3756.
[41] a)P. M. G. van Deursen, R. I. Koning, V. Tudor, M. A. Moradi, J. P. Patterson, A. Kros, N. Sommerdijk, A. J. Koster, G. F. Schneider, *Advanced Functional Materials* **2020**, 30, 1904468; b)M. Textor, N. de Jonge, *Nano Lett.* **2018**, 18, 3313.
[42] H. Wang, K. H. Nagamanasa, Y. J. Kim, O. H. Kwon, S. Granick, *ACS Nano* **2018**, 12, 8572.
